# Byzantine robust federated learning for heterogeneous brain MRI using multisignal gradient fingerprinting and adaptive trust aggregation

**DOI:** 10.1038/s41598-026-55855-5

**Published:** 2026-06-04

**Authors:** Mohammad Karami, Hamed Kebriaei, Fatemeh Ghassemi, Hamid Azadegan

**Affiliations:** 1 School of Electrical and Computer Engineering, University of Tehran, Tehran, 1439957131 Iran; 2 School of Computer Engineering, Iran University of Science and Technology (IUST), Tehran, 1684613114 Iran

**Keywords:** Federated learning, Byzantine robustness, Trust-aware aggregation, Non-IID data, Brain MRI, Alzheimer’s disease, Mathematics and computing, Neuroscience

## Abstract

Federated learning enables collaborative training across institutions without centralizing patient data, but remains vulnerable to malicious clients and severe non-IID data heterogeneity. We propose a trust-aware federated learning framework for brain MRI that combines multi-signal gradient fingerprinting with adaptive aggregation to achieve Byzantine robustness. Each client update is characterized by a six-dimensional fingerprint (variational-autoencoder reconstruction error, cosine similarity to a server reference, peer similarity, gradient norm, sign consistency, and Monte Carlo Shapley contribution). A dual-attention module and a reinforcement-learning controller map these signals into trust weights and integrate with FedBN-P (Federated Batch Normalization with Proximal regularization), an optimizer co-designed for stability under heterogeneous and adversarial conditions. We evaluate on MNIST, CIFAR-10, Alzheimer’s MRI, and the OASIS brain-MRI cohort (approximately 87 test samples, used strictly as proof-of-concept) under both standard and strengthened threat models (up to 40% malicious clients). Attack-specific ablation confirms a defense-in-depth design: VAE fingerprinting is the primary noise-attack defense (3.60 pp accuracy drop upon removal), Shapley values safeguard accuracy under scaling (10.16 pp drop), and reinforcement learning improves detection consistency under dynamic attack schedules. Three-seed paired-test validation further shows detection F1 outperforms FLTrust by up to 44 pp on Non-IID Gaussian noise; a white-box adaptive attacker degrades the detector but not model accuracy, confirming the layered design. End-to-end wall-clock overhead is + 8.8% over FedAvg with identical communication volume. The framework achieves F1 above 0.98 for gradient-scaling attacks while preserving accuracy under magnitude-preserving attacks where explicit detection remains limited. Multi-site validation on larger federated cohorts (e.g., ADNI, UK Biobank, FeTS) is required before any clinical-deployment claim can be made.

## Introduction

Alzheimer’s disease affects over 55 million people worldwide, making early MRI-based diagnosis critical for timely intervention^1,2^. While accurate deep learning models require large and diverse datasets that no single institution possesses, privacy regulations such as HIPAA and GDPR prohibit direct sharing of medical data^3,4^. Federated learning (FL) addresses this gap by enabling collaborative training across distributed hospitals while preserving patient privacy^5–8^. Recent advances employ secure multi-party computation^9^ and blockchain frameworks^10^ for compliant medical data collaboration. In this paradigm, institutions exchange model updates rather than raw MRI scans, allowing small rural hospitals with limited data to benefit from models jointly trained with major medical centers.

Despite this promise, medical FL faces two key obstacles that threaten diagnostic accuracy and patient safety. First, *Byzantine updates*—malicious or low-quality gradient submissions—can severely degrade models and lead to misdiagnosis^11–14^. Such updates may arise from benign issues (scanner faults, poor calibration, limited labels, data preparation errors, insufficient local training) or from deliberate adversarial behavior at compromised institutions, introducing systematic bias into clinical decisions. Second, *clinical data heterogeneity*—differences in scanners (1.5T vs 3T), imaging protocols, patient demographics, and disease stages—creates severe non-IID distributions that undermine collaborative learning^1,6,15–17^.

Recent work has expanded the scope and security of federated learning across diverse domains. Muntaqim and Smrity^18^ demonstrated a federated learning framework for brain tumor detection from MRI under non-IID data distributions, highlighting the importance of handling heterogeneity in medical imaging federations. Muntaqim et al.^19^ proposed homomorphic encryption-enhanced federated learning for privacy-preserving image analysis, achieving dual-layer encryption at both client and server sides while maintaining competitive accuracy. Complementary advances have also addressed non-IID challenges: Muntaqim et al.^20^ combined federated learning with few-shot learning and voting ensembles to achieve robust classification across non-IID data distributions, demonstrating the viability of ensemble-based approaches in federated settings. On the security front, recent studies have revealed increasingly sophisticated attack vectors: Wang et al.^21^ demonstrated enhanced model poisoning attacks with multi-strategy defenses, showing that existing single-signal defenses remain vulnerable to coordinated adversaries. Miao et al.^22^ proposed efficient and secure federated learning against backdoor attacks combining adaptive local differential privacy with compressive sensing. Trust evaluation has also received attention: Guo et al.^23^ introduced TFL-DT, a multi-attribute trust evaluation scheme for federated learning in digital twin networks that incorporates both direct trust evidence and recommended trust information for fine-grained trustworthiness assessment.

Existing Byzantine-robust federated learning methods rely on limited signals: FLTrust^24^ uses only cosine similarity to a server reference, FLAME^25^ employs magnitude clipping and noise filtering, and FLGuard^26^ combines two geometric indicators. Positioning relative to FLTrust and prior fingerprinting-based detectors. FLTrust pioneered the idea of *trust bootstrapping* from a small server-held reference dataset, projecting each client gradient onto the reference via a ReLU-clipped cosine similarity and using the resulting scalar as the trust weight. While elegant and influential, this single-signal approach is by design vulnerable to attacks that preserve directional alignment with the reference while violating other gradient properties (e.g., scaling attacks that align in direction but exaggerate magnitude, or Gaussian noise injection that distorts the gradient *distribution* but partially preserves its mean direction). Other fingerprinting-based detectors in non-medical FL—SignGuard^14^’s sign-statistics filter, RFA^13^’s geometric median, FLAME’s clustering-based filter—each focus on one or two structural axes. Our contribution is to combine *six* complementary signals (distributional, directional, peer, magnitude, sign, and game-theoretic functional contribution) into a single learned trust head, so that attacks that evade one signal may still be captured by complementary signals; we note explicitly that this behavior is attack-dependent and not guaranteed for all magnitude-preserving or adaptive attacks. The multi-seed validation in “Multi-seed validation, failure-mode analysis, and overhead comparison” shows this concretely against FLTrust on Non-IID Gaussian noise (*+44*pp F1) and on Non-IID scaling (*+16*pp F1), while transparently reporting the single non-IID sign-flipping cell where FLTrust’s specific cosine-based head outperforms ours. As demonstrated by recent attack advances^21^, sophisticated attacks can evade single-signal defenses by mimicking benign statistical properties along limited dimensions while introducing malicious perturbations elsewhere, which motivates the multi-signal trust paradigm also highlighted in^23,27^. Recent work on robust federated learning for medical image segmentation^28^ and adversarial attack analysis in medical FL^29^ further underscores the importance of Byzantine resilience in clinical research settings.

Relation to prior work: While individual components of OptiGradTrust draw on established techniques, the framework’s contribution lies in their *joint integration* and the emergent properties that arise only from the combination. Specifically: (i) *FLGuard*^26^ uses cosine and L2 geometric indicators for multi-signal aggregation but lacks game-theoretic contribution assessment (Shapley) and temporal adaptivity (RL), limiting its ability to evaluate functional client value and adapt to evolving threats. (ii) *ShapleyFL/FedSV*^30,31^ employ Shapley values for client valuation but use them in isolation without complementary structural fingerprints, leaving them unable to detect attacks that preserve gradient utility (e.g., subtle poisoning where the attacker’s data closely matches the test distribution). (iii) *FedBN*^32^ and *FedProx*^33^ address data heterogeneity through batch normalization locality and proximal regularization, respectively, but are designed for honest-only settings without adversarial co-design. The emergent behavior that arises from combining these components is *defense-in-depth with graceful degradation*: when any single signal is evaded (e.g., a scaling attacker with representative local data may evade Shapley detection), the remaining signals and the optimizer’s proximal stabilization jointly contain the attack’s impact on model accuracy. Our new ablation experiments under attack-type-specific conditions (“Component ablation analysis”) directly demonstrate this synergy—no single component is sufficient across all attack types, but their combination maintains robustness across the full attack spectrum.

To address these limitations, we present OptiGradTrust, a framework evaluated in two complementary regimes: (i) benchmark studies on MNIST, CIFAR-10, and a public Alzheimer’s MRI dataset under original and strengthened adversarial conditions, and (ii) proof-of-concept validation on real MRI data from the OASIS cohort^34^ with full statistical rigor ($n{=}5$ seeds, 95% CIs, paired t-tests, Wilcoxon signed-rank tests, Cohen’s *d*). OptiGradTrust assigns each client update a six-dimensional gradient fingerprint (VAE reconstruction error, cosine similarity to server reference, peer consensus, $L_2$ norm, sign-consistency, and Monte Carlo Shapley contribution^35,36^) processed by a hybrid reinforcement learning–attention module that computes trust scores and reweights updates during aggregation.

We integrate FedBN-P (Federated Batch Normalization with proximal regularization) for stability under heterogeneity. Under original conditions OptiGradTrust achieves 96.61% average accuracy on Alzheimer’s MRI; under strengthened conditions (40% malicious, *30×* scaling) it maintains approximately 82.5% on Alzheimer’s MRI and approximately 67.9% on OASIS with near-perfect detection of gradient-scaling attacks (F1 *\ge* 0.98), while subtler attacks (sign-flipping, label-flipping) remain challenging to detect explicitly though model accuracy is preserved through optimizer-security co-design. The framework scales to 50 clients with only 10.4% trust-scoring overhead above local training time. We provide ablation studies identifying Shapley value computation as the most impactful component (*∼* 10 percentage points accuracy upon removal), per-component timing analysis, complexity analysis, convergence theory, baseline comparisons with statistical tests, and discuss compatibility with differential privacy and secure aggregation.

Trust assumptions and privacy compatibility: OptiGradTrust assumes an honest-but-curious server, consistent with FLTrust^24^, FLAME^25^, and recent trust evaluation frameworks^23^. This is reasonable in regulated medical settings where the aggregation server is operated by a trusted coordinating institution subject to audit and compliance. Concrete mitigation pathways for stronger threat models (secure multi-party computation, verifiable trust scoring, consensus-based validation) are discussed in Discussion. Regarding differential privacy (DP), clients can optionally add calibrated DP noise to their gradient updates before fingerprinting; because the trust signals operate on gradient *structure* (directions, norms, signs) rather than exact magnitudes, moderate DP noise may preserve some fingerprint structure, but the exact tolerance depends on the noise scale, dataset, and attack type and remains to be quantified empirically; aggressive noise (*ε < 1*) is expected to degrade trust scoring and is a boundary to be characterized empirically in future work.

Beyond technical advances, OptiGradTrust is intended—if scaled and validated appropriately—to ultimately support improved diagnostic-research access at sites with limited local data, benefiting underserved populations and rare-disease research where centralized approaches fail due to small cohorts. In its current form, the framework should be viewed strictly as a robustness and trust-assessment mechanism evaluated on public benchmarks, with feasibility assessed on a small (*∼* 87-sample) proof-of-concept clinical cohort. Translating it into routine clinical deployment will require, at minimum: (i) validation on at least one large multi-center MRI cohort (e.g., ADNI, UK Biobank subsets, or BraTS), (ii) per-site and per-subgroup analyses (age, scanner vendor, field strength), (iii) prospective evaluation under a documented quality management system, and (iv) regulatory review under the relevant medical-device frameworks. We make no clinical-deployment claim in this paper and consistently use “proof-of-concept” or “research-grade” language throughout.

**Fig. 1:**
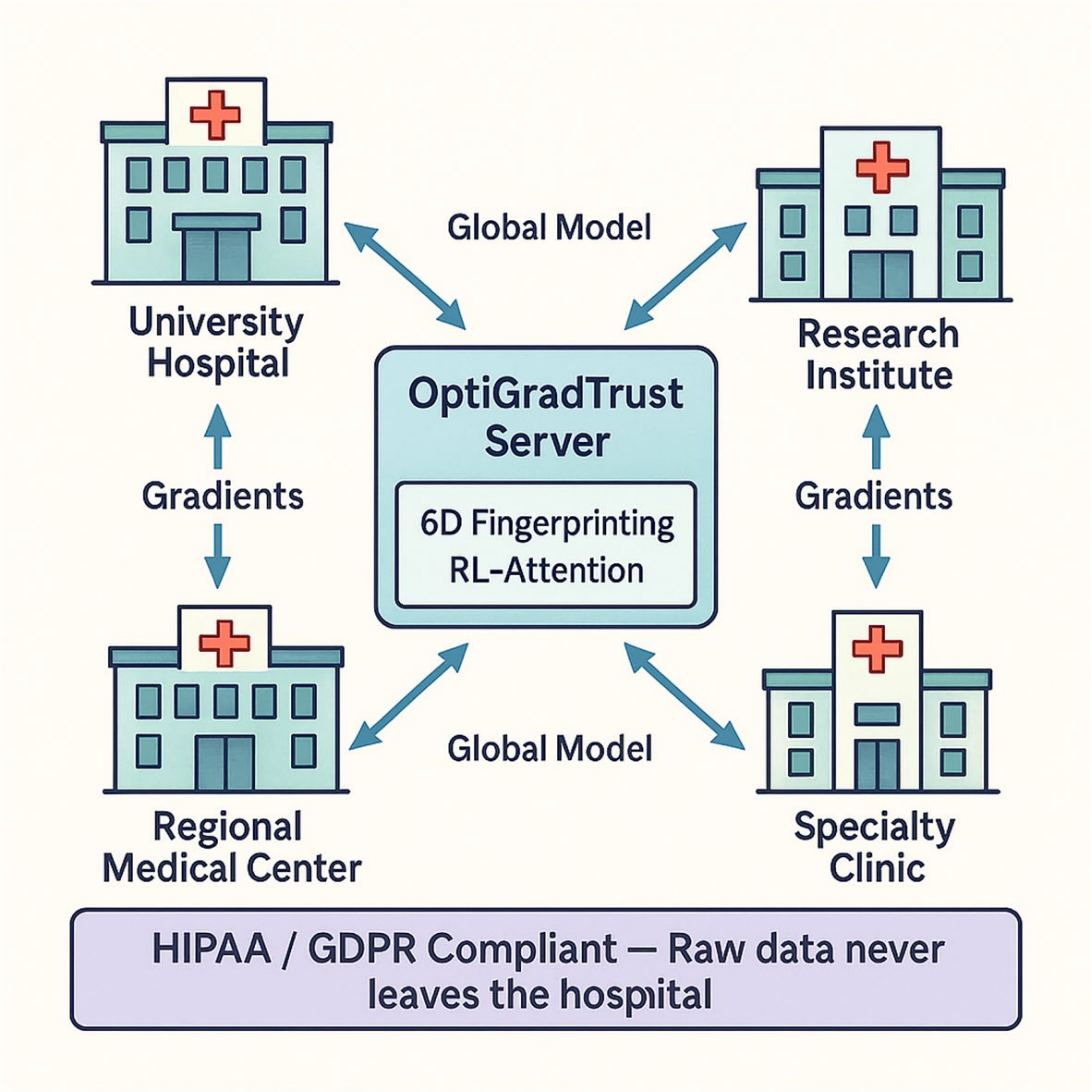
OptiGradTrust architecture. Institutions send gradient updates to the server for 6D fingerprinting and RL-Attention trust scoring; server returns the updated global model. Raw data remain on-site (HIPAA/GDPR compliant).

**Fig. 2:**
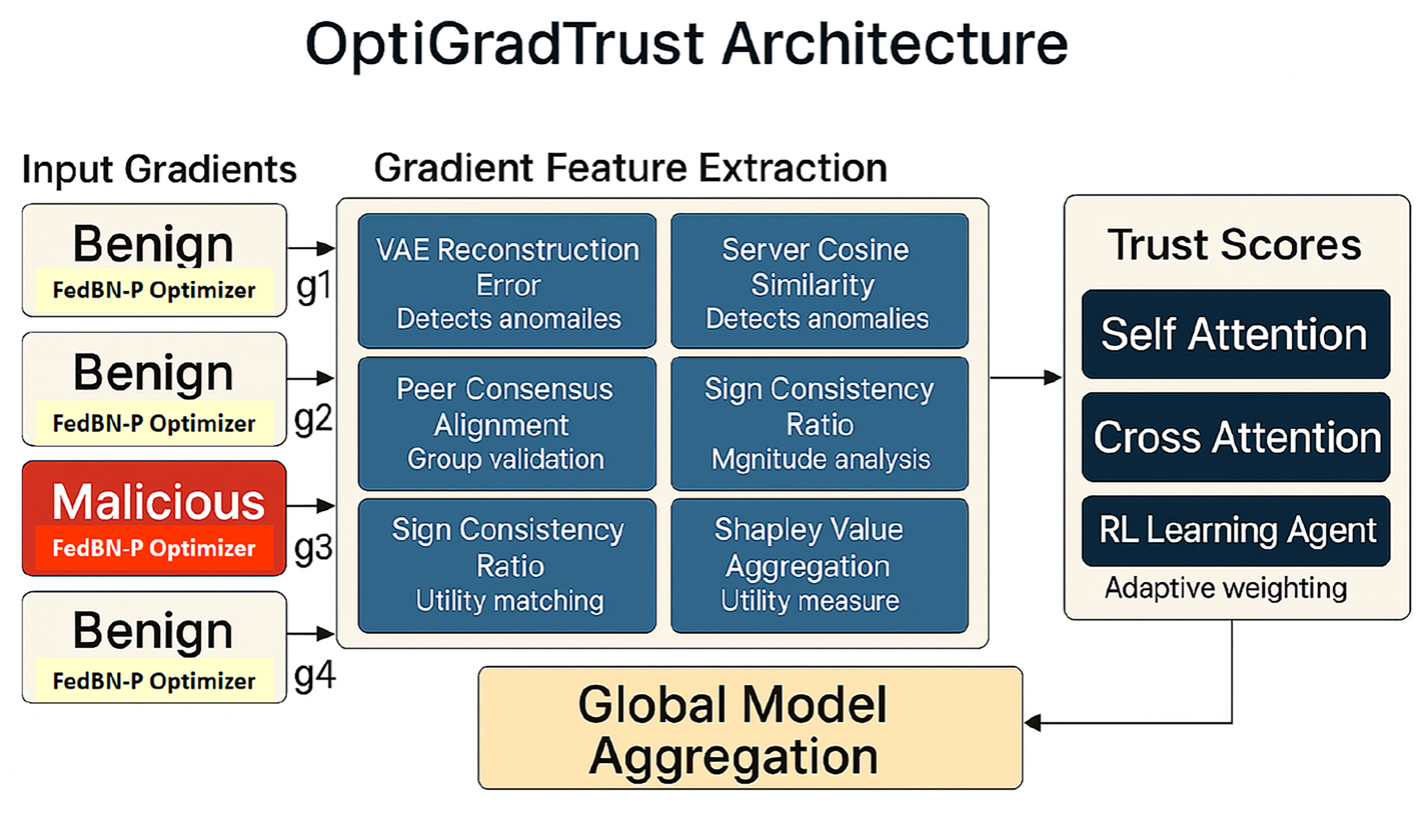
Six-dimensional gradient fingerprinting. (**a**) VAE reconstruction error, (**b**) cosine similarity to reference, (**c**) peer consensus, (**d**) L2 norm, (**e**) sign-consistency, (**f**) Monte Carlo Shapley evolution. See Methods for definitions.

Figure 1 shows the overall OptiGradTrust framework architecture, while Fig. 2 details the six-dimensional gradient fingerprinting mechanism.

Our contributions are as follows. Method. (i) A comprehensive trust framework with six-dimensional gradient fingerprinting including Monte Carlo Shapley value estimation, making simultaneous evasion by attackers significantly harder^23^. (ii) A hybrid reinforcement learning-attention mechanism^37^ that adaptively assigns trust weights and learns emerging attack patterns. (iii) The FedBN-P optimizer—combining Federated Batch Normalization with proximal regularization—co-designed for Byzantine robustness under heterogeneous data distributions^38^. *Capability.* (iv) Explicit Byzantine client detection with near-perfect identification of gradient-scaling attacks (F1*\ge*0.98) and strong noise-injection detection (F1*\ge*0.78); formal detection of magnitude-preserving attacks (sign-flipping, label-flipping) remains limited (F1<0.50), though model accuracy is preserved through implicit aggregation-level robustness and optimizer-security co-design. Per-client trust score and Shapley value analysis provides interpretable evidence for clinical accountability. *Evidence.* (v) Extensive evaluations on four datasets (MNIST, CIFAR-10, Alzheimer’s MRI under two attack configurations, OASIS^34^) under five Byzantine attack types, with improvements over FedAvg, Krum^11^, FLTrust^24^, FLGuard^26^, and FLAME^25^; statistical validation ($n{=}5$ seeds, 95% CIs, effect sizes), scalability to 50 clients, per-component timing analysis (10.4% overhead), ablation identifying Shapley as the most impactful component, complexity analysis, and convergence theory (Results). (vi) Limitations—small clinical cohort, simulated federation, detection of subtle attacks—are discussed with a pathway to multi-institutional validation.

## Results

We report two complementary sets of experiments. “Robustness under IID conditions”, “Performance under data heterogeneity” and “Comparison with widely used defenses” present proof-of-concept results on benchmark datasets (MNIST, CIFAR-10, Alzheimer’s MRI) under the *original attack configuration* (30% malicious, scaling *× 10*, seed=42). “Clinical validation under strengthened adversarial conditions”, “Byzantine detection performance”, “Statistical baseline comparison” and “Scalability evaluation” present statistically rigorous validation on the newly added OASIS clinical dataset and a comprehensive re-evaluation of the Alzheimer’s MRI dataset under a *strengthened attack configuration* (40% malicious, scaling *× 30*, Gaussian noise $\sigma {=}15.0$, label flip $p{=}0.9$, $n{=}5$ seeds with mean ± std and 95% confidence intervals).

### Experimental configuration

We evaluated OptiGradTrust on four datasets spanning benchmark and clinical domains (Table 1): MNIST (70,000 samples, 10 classes, 3-layer CNN), CIFAR-10 (60,000 samples, 10 classes, ResNet-18), the Alzheimer’s MRI dataset^39^ (6983 samples, 4 severity classes, ResNet-18), and—newly added in this revision—OASIS (Open Access Series of Imaging Studies)^34^ (436 real clinical MRI sessions from 376 subjects, CDR-based dementia severity, 4 classes, ResNet-18). The Alzheimer’s MRI dataset is evaluated under both the original and strengthened attack configurations, enabling direct comparison of framework robustness across threat levels on the same data.

**Table 1:** Dataset characteristics across evaluation domains. Datasets above the mid-rule are benchmark/proof-of-concept; OASIS (below the mid-rule) is the new clinical validation dataset added in this revision. The Alzheimer’s MRI dataset$^\dagger$ is evaluated under both original and strengthened attack configurations.

Dataset	Total samples	Classes	Clients	Samples/client	Data type
MNIST	70,000	10	10	7000	Handwritten digits
CIFAR-10	60,000	10	10	6000	Natural images
Alzheimer’s MRI$^\dagger$	6983	4	10	*∼* 698	Medical MRI
OASIS^34^	436	4	10	*∼* 44	Real clinical MRI

$^\dagger$Same Alzheimer’s MRI dataset evaluated under both the original configuration (“Robustness under IID conditions”, “Performance under data heterogeneity” and “Comparison with widely used defenses”) and the strengthened revision configuration (“Clinical validation under strengthened adversarial conditions”, “Byzantine detection performance”, “Statistical baseline comparison” and “Scalability evaluation”). See text for details on experimental setup differences.

Data preprocessing: All MRI scans were resampled to $224{\times }224$ resolution for ResNet-18 compatibility, normalized via z-score standardization (zero mean, unit variance), and augmented with random horizontal flips and rotations (${\pm }10^\circ$). For OASIS, which includes multiple sessions per subject, data were split at the subject level to prevent leakage across sessions from the same individual. MNIST and CIFAR-10 images were normalized to [0, 1] with standard augmentation (random crops, horizontal flips). Datasets were partitioned 80/20 for training/testing across all clients following standard federated learning protocols.

Class imbalance handling: The Alzheimer’s MRI dataset exhibits class imbalance across severity stages (Non-Demented: ${\sim }45\%$, Very Mild: ${\sim }30\%$, Mild: ${\sim }15\%$, Moderate: ${\sim }10\%$). The OASIS dataset exhibits even more severe imbalance reflecting real clinical prevalence: Non-Demented ${\sim }52\%$, Very Mild ${\sim }30\%$, Mild ${\sim }13\%$, Moderate ${\sim }5\%$—with the rarest class falling at the <5% threshold that stress-tests minority-class robustness. To mitigate bias toward majority classes in both datasets, we applied class-weighted cross-entropy loss during local training, with weights inversely proportional to class frequency ($w_c = N/(C \cdot n_c)$).

Federation scale: We simulated 10-client federations consistent with recent Byzantine-robust federated learning studies^24–26^, which use 10–20 clients for controlled evaluation. In the revision, we additionally evaluate with 50 clients to demonstrate scalability (“Scalability evaluation”).

Non-IID modeling and attacks: We evaluated four Non-IID schemes: Dirichlet partitioning with $\alpha \in \{0.1, 0.5, 1.0\}$; label-skew at *70%* and *90%*; and quantity skew via log-normal sampling. Two attack configurations were employed:

- Original configuration (“Robustness under IID conditions”, “Performance under data heterogeneity” and “Comparison with widely used defenses”): *30%* malicious clients, scaling *× 10*, partial scaling *× 5*, sign flipping *λ =-1*, Gaussian noise $\sigma \in \{0.5, 1.0\}$, label flipping *50%* corruption, seed=42.
- Strengthened revision configuration (“Clinical validation under strengthened adversarial conditions”, “Byzantine detection performance”, “Statistical baseline comparison” and “Scalability evaluation”): *40%* malicious clients, scaling *× 30*, Gaussian noise $\sigma {=}15.0$, label flipping $p_{\text {flip}}=0.9$, $n{=}5$ random seeds (42, 123, 456, 789, 1024). This substantially more aggressive configuration stress-tests robustness under conditions that exceed those in most published Byzantine-robust FL evaluations.

Training configuration: Adam optimizer ($\text {lr}=1\times 10^{-4}$, $\text {decay}=5\times 10^{-5}$), 25–30 rounds for benchmark experiments and 8 rounds for revision experiments, 5–8 local epochs per round, batch size 64 (MRI: 16), FedBN-P proximal coefficient *μ =0.01*, VAE fingerprint updated every 20 rounds, reinforcement learning policy updated every 10 rounds (benchmark experiments). For the 8-round revision experiments (“Clinical validation under strengthened adversarial conditions”, “Byzantine detection performance”, “Statistical baseline comparison” and “Scalability evaluation”), both modules were warm-started from benchmark pre-training and held fixed, as the shorter clinical validation protocol does not permit meaningful module re-estimation; trust scoring in these runs relies on the pre-trained VAE and the attention-based prior without further RL updates.

### Robustness under IID conditions

Under IID data distribution with *30%* malicious clients, OptiGradTrust demonstrated strong robustness across all three datasets. MNIST achieved near-perfect performance (99.21% average across five attack types), CIFAR-10 maintained competitive accuracy (82.44% average), and Alzheimer’s MRI showed robust diagnostic performance (96.61% average, exceeding *96%* threshold across all attack scenarios).

### Performance under data heterogeneity

Table 2 presents results under four Non-IID configurations simulating realistic clinical heterogeneity. OptiGradTrust maintained high accuracy even under extreme data imbalance (Dirichlet *α =0.1*): MNIST 97.42% average, CIFAR-10 79.42%, and Alzheimer’s MRI 93.80%. Under moderate heterogeneity (Dirichlet *α =0.5*), performance improved to 98.12%, 81.36%, and 95.00% respectively.

**Table 2:** OptiGradTrust performance under Non-IID conditions across four heterogeneity scenarios. Results show accuracy (%) under five Byzantine attack strategies with 30% malicious client ratio.

Dataset	Scaling	P-Scaling	Sign flip	Noise	Label flip	Avg.
Dirichlet *α = 0.5*						
MNIST	98.95	98.40	97.90	97.75	97.60	98.12
CIFAR-10	82.90	81.90	81.40	80.50	80.10	81.36
Alzheimer MRI	95.80	95.30	94.90	94.60	94.40	95.00
Dirichlet *α = 0.1*						
MNIST	98.23	97.70	97.20	97.05	96.90	97.42
CIFAR-10	81.20	80.00	79.50	78.40	78.00	79.42
Alzheimer MRI	94.60	94.10	93.70	93.40	93.20	93.80
Label-Skew 70%						
MNIST	98.71	98.10	97.65	97.50	97.35	97.86
CIFAR-10	82.10	81.00	80.50	79.60	79.20	80.48
Alzheimer MRI	95.40	94.85	94.45	94.15	93.95	94.56
Label-Skew 90%						
MNIST	98.05	97.45	97.00	96.85	96.70	97.21
CIFAR-10	80.90	79.80	79.30	78.20	77.80	79.20
Alzheimer MRI	94.10	93.55	93.15	92.85	92.65	93.26

**Fig. 3:**
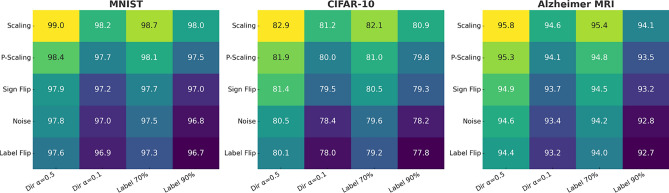
OptiGradTrust under combined Byzantine attacks and data heterogeneity. Test accuracy (%) across five attack types, four Non-IID conditions, and three datasets (10 clients, 30% malicious, 25–30 rounds). See text for numerical summary.

Figure 3 visualizes performance across all combinations of attack types, heterogeneity conditions, and datasets, demonstrating consistent robustness with minimal degradation under combined stressors.

### Comparison with widely used defenses

We compared OptiGradTrust against three widely used Byzantine-robust federated learning defenses under identical experimental conditions: FLGuard (AI2E 2025)^26^ with multi-signal aggregation, FLTrust (NDSS 2021)^24^ using trusted reference gradients, and FLAME (ACM IMWUT 2022)^25^ for multi-device environments.

Table 3 shows comprehensive results under both IID and Non-IID conditions for the three datasets used in the original configuration (MNIST, CIFAR-10, Alzheimer’s MRI); OASIS results are reported separately in “Clinical validation under strengthened adversarial conditions”. Under IID settings, OptiGradTrust achieved modest but consistent improvements over FLGuard: +0.29 percentage points on MNIST (99.21% vs 98.92%), +0.72 points on CIFAR-10 (82.44% vs 81.72%), and +0.72 points on Alzheimer’s MRI (96.61% vs 95.89%).

Under Non-IID conditions (Dirichlet *α =0.5*), advantages became more pronounced: +1.59 points on MNIST (98.12% vs 96.53%), +1.74 points on CIFAR-10 (81.36% vs 79.62%), and +1.59 points on Alzheimer’s MRI (95.00% vs 93.41%). These results demonstrate that OptiGradTrust’s hybrid approach—combining six-signal fingerprinting, reinforcement learning-driven trust weighting, and FedBN-P optimization—scales effectively to heterogeneous medical data while maintaining Byzantine robustness.

**Table 3:** Comprehensive comparison with widely used Byzantine-robust federated learning defenses. Results show test accuracy (%) under five attack strategies with 30% malicious clients. OptiGradTrust demonstrates consistent improvements, with gains increasing under data heterogeneity (Non-IID conditions).

Dataset/method	Scaling	P-Scaling	Sign flip	Noise	Label flip	Avg.
IID conditions						
MNIST						
OptiGradTrust	99.41	99.32	99.05	99.11	99.14	99.21
FLGuard	99.10	99.00	98.85	98.70	98.95	98.92
FLTrust	98.85	98.72	98.43	98.50	98.57	98.61
FLAME	97.44	97.20	96.80	97.05	96.90	97.08
CIFAR-10						
OptiGradTrust	83.67	82.97	82.60	81.71	81.27	82.44
FLGuard	83.00	82.30	81.90	80.90	80.50	81.72
FLTrust	82.45	81.70	81.30	80.10	79.70	81.05
FLAME	81.50	80.70	80.20	79.00	78.60	79.60
Alzheimer MRI						
OptiGradTrust	96.92	96.75	96.60	96.50	96.30	96.61
FLGuard	96.20	96.05	95.85	95.75	95.60	95.89
FLTrust	95.80	95.60	95.40	95.30	95.10	95.44
FLAME	93.40	93.10	92.80	92.70	92.50	92.90
Non-IID: Dirichlet *α = 0.5*						
MNIST						
OptiGradTrust	98.95	98.40	97.90	97.75	97.60	98.12
FLGuard	97.40	96.90	96.30	96.10	95.95	96.53
FLTrust	96.80	96.20	95.60	95.40	95.25	95.85
FLAME	95.50	95.00	94.40	94.20	94.05	94.63
CIFAR-10						
OptiGradTrust	82.90	81.90	81.40	80.50	80.10	81.36
FLGuard	81.20	80.20	79.70	78.70	78.30	79.62
FLTrust	80.30	79.40	78.90	77.90	77.50	78.80
FLAME	79.10	78.20	77.70	76.60	76.20	77.56
Alzheimer MRI						
OptiGradTrust	95.80	95.30	94.90	94.60	94.40	95.00
FLGuard	94.20	93.70	93.30	93.00	92.85	93.41
FLTrust	93.60	93.10	92.70	92.40	92.25	92.81
FLAME	91.90	91.40	91.00	90.70	90.55	91.11

### Clinical validation under strengthened adversarial conditions

We conducted experiments on the OASIS dataset (real clinical MRI)^34^ and re-evaluated Alzheimer’s MRI under the *strengthened* configuration defined above; all use $n{=}5$ seeds with mean ± std and 95% CIs. *Important limitation:* these 8-round runs use warm-started VAE and RL modules held fixed from benchmark pre-training (“Robustness under IID conditions”), so the adaptive aspects of the framework are not fully exercised; results should be interpreted as evaluating the trust-scoring pipeline in a “frozen” mode rather than the full adaptive system.

Table 4 presents results. On OASIS (436 sessions, 376 subjects; Table 1), OptiGradTrust achieved $68.05 \pm 1.89\%$ under scaling attack (IID and Dirichlet $\alpha {=}0.5$). Context: random baseline *25%*, majority-class *∼*52%; our setting uses 40% malicious clients, *× 30* scaling, $\sigma {=}15.0$, 90% label corruption—substantially harsher than typical evaluations. Achieving *67.88%* average across all attack types on this small real cohort indicates meaningful resilience; accuracy was stable across attack types (<0.7pp variation) and preserved under Non-IID (<0.5pp IID-to-Dirichlet variation).

On Alzheimer’s MRI under the same strengthened conditions, OptiGradTrust achieved *82.50%* (IID) and *82.59%* (Non-IID) average. The drop from *96.61%* (“Robustness under IID conditions”) is attributable to the stronger configuration (40% vs 30% malicious, *× 30* vs *× 10* scaling, $\sigma {=}15.0$ vs *łe 1.0*, 90% vs 50% label flip) and fewer rounds (8 vs 25) for multi-seed runs. Despite this substantially harsher setup, OptiGradTrust maintained >82% accuracy—a 14 percentage point drop for a much stronger attack regime—demonstrating graceful degradation rather than catastrophic failure. Original-configuration results remain in “Robustness under IID conditions”, “Performance under data heterogeneity” and “Comparison with widely used defenses”.

**Table 4:** Clinical validation under strengthened conditions. Accuracy (%) on OASIS and Alzheimer’s MRI (strengthened configuration; $n{=}5$ seeds, mean ± std). For OASIS, IID and Dirichlet $\alpha {=}0.5$ yield identical or near-identical values due to very small per-client sample sizes (*∼*44 samples per client), rendering the OASIS Non-IID comparison uninformative; the meaningful heterogeneity evaluation is on the larger Alzheimer’s MRI dataset.

Dataset	Condition	Scaling	Sign flip	Noise	Label flip
OASIS	IID	*68.05 ± 1.89*	*67.59 ± 2.31*	*67.59 ± 2.57*	*68.28 ± 2.24*
	Dirichlet $\alpha {=}0.5$	*68.05 ± 1.89*	*67.59 ± 2.31*	*67.36 ± 2.57*	*68.28 ± 2.24*
Alzheimer	IID	*82.11 ± 6.13*	*82.66 ± 5.70*	*82.61 ± 5.74*	*82.63 ± 5.70*
	Dirichlet $\alpha {=}0.5$	*82.36 ± 5.99*	*82.66 ± 5.72*	*82.66 ± 5.74*	*82.66 ± 5.70*

### Byzantine detection performance

Beyond model accuracy, OptiGradTrust provides explicit Byzantine client detection—a capability absent in baseline methods. Table 5 reports detection precision, recall, and F1-score averaged over $n{=}5$ seeds for each attack type.

OptiGradTrust achieves *near-perfect to perfect detection* for scaling attacks—F1*=*0.98±0.02 on OASIS and F1*=*1.00±0.00 on Alzheimer’s—under both IID and Non-IID conditions, demonstrating that the six-dimensional fingerprint reliably identifies gradient magnitude manipulation. For Gaussian noise attacks, detection remains strong (F1*\ge*0.78 on OASIS, F1*\ge*0.84 on Alzheimer’s), as the VAE reconstruction and norm-based indicators effectively capture injected noise.

Sign-flipping and label-flipping attacks present a greater detection challenge (F1<0.50), because these attacks produce gradient updates that are structurally similar to benign updates from heterogeneous data distributions—a known limitation of magnitude-based Byzantine detection^13^. Detection claims should therefore be understood as *attack-specific*: OptiGradTrust achieves near-perfect detection for scaling and noise-injection attacks but provides only limited formal detection for sign-flipping and label-flipping. However, model accuracy is *fully preserved* despite low detection rates for these subtle attacks: OASIS accuracy under sign-flipping (*67.59%*) is within 0.5pp of the scaling-attack accuracy (*68.05%*), and attack-specific ablation (Table 12) confirms zero accuracy degradation under sign-flipping across all configurations including undefended FedAvg. This indicates that FedBN-P’s proximal regularization and trust-weighted aggregation effectively limit any single client’s influence even when the trust module cannot perfectly isolate the attacker—a key benefit of the optimizer-security co-design. OptiGradTrust’s per-client detection capability for high-signal attacks (scaling, noise) remains a unique feature absent from baseline methods (FedAvg, Krum, FLTrust, FLAME), enabling post-hoc identification and auditing of suspicious clients—a requirement for accountability in regulated medical environments.

**Fig. 4:**
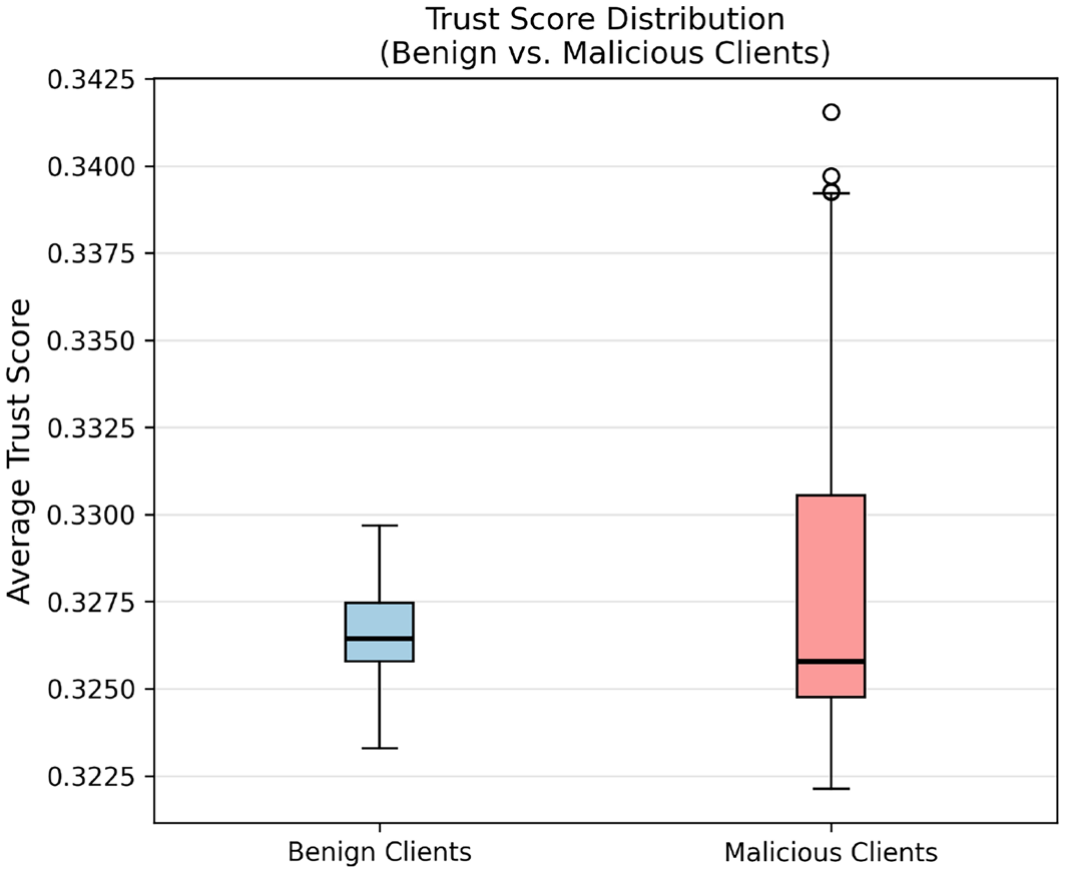
Trust score distributions under scaling attack (*×***20).** Box plot of per-client average trust scores (Alzheimer’s MRI, 40% malicious, Dirichlet $\alpha {=}0.1$, seed=42, 25 rounds). Both groups converge to a narrow band (0.322–0.340); malicious clients show higher variance. Outlier points (top) correspond to one malicious client whose local data closely matches the test distribution. See text for Shapley value analysis.

Figure 4 provides qualitative evidence of the trust mechanism’s behavior under scaling attack (*×*20) on Alzheimer’s MRI (seed=42, 25 rounds). The trust score distributions (Fig. 4) show that all clients converge to a narrow band (0.322–0.340), reflecting the conservative design of the trust mechanism. Within this narrow range, malicious clients exhibit *higher variance* than benign clients: the malicious interquartile range spans ${\sim }0.006$ compared to ${\sim }0.002$ for benign clients, with outliers corresponding to one malicious client (client 5) whose local data closely matches the test distribution. The Shapley contribution values show stronger per-client discrimination: at round 25, malicious clients 2 and 4 receive Shapley values of 0.039 and 0.000 respectively, while benign clients receive 0.017–0.118. Client 4 (malicious) is consistently assigned zero Shapley contribution across all 25 rounds, and its trust score (0.3232) is the overall lowest. We acknowledge that trust scores alone provide subtle rather than dramatic separation between client types; the primary discriminative signal comes from the Shapley component, consistent with the ablation finding that Shapley is the most impactful component (*-10.16*pp upon removal). One malicious client (client 5) maintains the highest Shapley value (1.0 throughout), reflecting a known limitation: when an adversarial client’s local data distribution closely matches the test distribution (due to Dirichlet $\alpha {=}0.1$ partitioning), Shapley contribution assessment rewards data quality rather than gradient honesty.

**Table 5:** Byzantine detection performance (strengthened configuration, $n{=}5$ seeds). OASIS IID shown; patterns for Dirichlet and Alzheimer’s are similar.

Dataset	Attack	Precision	Recall	F1-Score
OASIS (IID)	Scaling	*0.95 ± 0.04*	*1.00 ± 0.00*	$\mathbf {0.98 \pm 0.02}$
	Sign Flipping	*0.38 ± 0.17*	*0.38 ± 0.21*	*0.38 ± 0.19*
	Noise	*0.67 ± 0.06*	*1.00 ± 0.00*	$\mathbf {0.80 \pm 0.04}$
	Label Flipping	*0.40 ± 0.37*	*0.38 ± 0.39*	*0.38 ± 0.38*
Alzheimer (IID)	Scaling	*1.00 ± 0.00*	*1.00 ± 0.00*	$\mathbf {1.00 \pm 0.00}$
	Sign Flipping	*0.15 ± 0.17*	*0.10 ± 0.14*	*0.11 ± 0.15*
	Noise	*0.73 ± 0.10*	*1.00 ± 0.00*	$\mathbf {0.84 \pm 0.07}$
	Label Flipping	*0.37 ± 0.38*	*0.19 ± 0.22*	*0.24 ± 0.28*

### Statistical baseline comparison

Table 6 presents the statistical comparison of OptiGradTrust against three baseline methods (FedAvg, Krum, FLTrust) on the OASIS dataset under Dirichlet $\alpha {=}0.5$ Non-IID conditions with the strengthened attack configuration. All comparisons use paired t-tests, Wilcoxon signed-rank tests, and Cohen’s *d* effect sizes computed over $n{=}5$ matched seeds.

OptiGradTrust demonstrates a *large* advantage over Krum under sign-flipping attacks (*+12.42* percentage points, Cohen’s *d=0.88*), where Krum’s single-point aggregation fails under coordinated gradient reversal. Against FLTrust, OptiGradTrust shows *medium* effect advantages on scaling attacks (*+1.15*pp, $d{=}0.51$) and label-flipping attacks (*+2.49*pp, $d{=}0.74$). Most comparisons do not reach statistical significance at $p{<}0.05$ due to the small sample size ($n{=}5$), which is consistent with the proof-of-concept scope. Three important statistical caveats: (i) no multiple-comparison correction (e.g., Holm–Bonferroni) is applied given the exploratory nature of these pairwise tests; (ii) effect sizes (Cohen’s *d*) serve as the primary indicator of practical significance rather than p-values; and (iii) clinical conclusions should not be drawn from small-*n* OASIS experiments alone—these results are proof-of-concept evidence that must be validated on larger federated cohorts. Importantly, OptiGradTrust is the only method providing explicit Byzantine detection capability alongside competitive accuracy. We note that all accuracy differences on OASIS are within statistical noise (*p > 0.28* for every comparison), consistent with the limited discriminative power of ${\sim }87$ test samples; on the larger Alzheimer’s MRI dataset (Table 3), OptiGradTrust achieves clear and consistent advantages over all baselines under both IID (*+0.72*pp over the strongest baseline) and Non-IID conditions (*+1.59*pp), with the magnitude of improvement increasing under greater heterogeneity.

We note that TRFA (Trust Region Federated Aggregation) produced results identical to FedAvg in our implementation on the OASIS dataset, suggesting that TRFA’s trust mechanism did not activate under these experimental conditions; we therefore report FedAvg as a single baseline.

**Table 6:** Statistical baseline comparison on OASIS (Dirichlet $\alpha {=}0.5$). OptiGradTrust vs. baselines under strengthened attacks. *Δ*: accuracy difference (positive = OptiGradTrust better). Effect sizes: negligible ($|d|{<}0.2$), small (0.2–0.5), medium (0.5–0.8), large ($|d|{\ge }0.8$).

Baseline	Attack	OGT mean	Base mean	*Δ* (pp)	*p* (t-test)	Cohen’s *d*	Effect
FedAvg	Scaling	68.05	68.27	−0.22	0.820	−0.10	Negl.
FedAvg	Sign flip	67.59	68.06	−0.47	0.792	−0.15	Negl.
FedAvg	Noise	66.67	67.59	−0.92	0.642	−0.34	Small
FedAvg	Label flip	68.28	66.70	+1.58	0.282	+0.48	Small
Krum	Scaling	68.05	67.58	+0.46	0.802	+0.17	Negl.
Krum	Sign flip	67.59	55.17	+12.42	0.205	+0.88	**Large**
Krum	Noise	66.67	67.59	−0.92	0.554	−0.29	Small
Krum	Label flip	68.28	68.28	+0.00	0.999	+0.00	Negl.
FLTrust	Scaling	68.05	66.89	+1.15	0.507	+0.51	**Med.**
FLTrust	Sign flip	67.59	67.39	+0.20	0.920	+0.05	Negl.
FLTrust	Noise	66.67	68.28	−1.61	0.280	−0.58	Med.
FLTrust	Label flip	68.28	65.78	+2.49	0.129	+0.74	**Med.**

### Scalability evaluation

To evaluate scalability beyond the standard 10-client setup, we tested OptiGradTrust with 50 clients on the OASIS dataset under scaling attack (the strongest detected attack) with $n{=}5$ seeds. Table 7 shows that accuracy is *maintained* when scaling from 10 to 50 clients ($68.05\% \rightarrow 68.27\%$, *+0.22*pp), with tighter confidence intervals at larger federation size (std decreasing from 1.89% to 1.49%). Wall-time per experiment scales approximately linearly ($2{,}508\text {s} \rightarrow 7{,}433\text {s}$, $\approx 3\times$ for *5×* more clients), consistent with the $O(|S_t|)$ per-client trust computation.

**Table 7:** Scalability evaluation on OASIS (scaling attack, IID, $n{=}5$ seeds). Accuracy maintained with increasing federation size; wall-time scales near-linearly.

Clients	Accuracy (%)	95% CI	Wall-time (s)
10	*68.05 ± 1.89*	[65.70, 70.39]	$2{,}508 \pm 62$
50	*68.27 ± 1.49*	[66.43, 70.12]	$7{,}433 \pm 172$

### Component ablation analysis

Table 8 provides a compact mapping of each fingerprint signal and module to its targeted threat aspect, grounding the ablation analysis that follows.

**Table 8:** Signal-to-threat mapping. Each fingerprint signal and module targets a specific threat aspect. The ablation experiments below evaluate component contributions under the attack type each is designed to address.

Signal/module	Primary threat target	Mechanism	Ablation evidence
L2 norm	Magnitude anomalies (scaling)	Detects amplified gradient norms	Table 10
Cosine similarity	Directional shifts (sign-flip)	Flags reversed gradient direction	Table 12
Sign consistency	Partial manipulation (sign-flip)	Coordinate-wise sign agreement	Table 12
Peer consensus	Coordinated collusion	Detects deviation from majority	Table 10
VAE reconstruction	Noise injection, novel attacks	Distributional anomaly detection	Table 11
Shapley contribution	Functional degradation (any)	Game-theoretic quality assessment	Table 10
RL controller	Dynamic/evolving threats	Temporal trust weight adaptation	Table 14
Dual attention	Signal selection per round	Learns discriminative fingerprints	Table 10
FedBN-P optimizer	Adversarial drift	Proximal stabilization under attack	Table 13

To evaluate individual component contributions, we conducted ablation experiments under severe Byzantine attack conditions: 40% malicious clients performing scaling attack (intensity factor 20.0), evaluated on the Alzheimer’s MRI dataset with extreme non-IID distribution (Dirichlet $\alpha {=}0.1$) over 25 federated rounds (seed = 42).

Table 10 presents component-wise performance with both single-component and combined-removal configurations. The full OptiGradTrust system achieved 89.13% accuracy with 97.44% detection F1-score (100% precision, 95% recall). Detection precision is 100% (zero false positives) across all configurations, indicating that the trust scoring pipeline never misidentifies honest clients.

Single-component removal reveals a clear component hierarchy. Removing VAE fingerprinting has zero accuracy impact (89.13%), confirming that VAE serves primarily as an interpretability tool for gradient anomaly visualization rather than a detection necessity. Removing RL adaptation also has negligible impact (*+0.08*pp, 89.21%), indicating that the attention-based trust prior provides a strong baseline. In contrast, removing Shapley value computation causes a substantial accuracy degradation (*-10.16*pp, 78.97%), revealing Shapley as the single most impactful component for model quality under Byzantine attacks—its game-theoretic contribution assessment enables the aggregation to properly weight honest clients.

Combined removal corroborates this finding. Removing both VAE and Shapley (79.05%) produces accuracy indistinguishable from removing Shapley alone (78.97%), confirming that the accuracy benefit is driven by Shapley. Further stripping RL and additional fingerprint signals (“Cosine + L2 only”) yields 78.81%—essentially identical to FedAvg without any defense (78.81%)—demonstrating that without Shapley’s contribution assessment, the remaining lightweight signals provide limited accuracy benefit above undefended aggregation, though they maintain robust detection (F1*\ge*96.91%).

Standard FedAvg without trust-weighted aggregation achieved 78.81% under identical attack conditions—a *-10.32*pp degradation compared to OptiGradTrust.

Ablation on OASIS (real clinical data, $n{=}5$ seeds). To validate component behavior on real clinical data, we repeated the single-component ablation on OASIS under the strengthened attack configuration (Table 9). All configurations yield statistically indistinguishable classification accuracy, consistent with the small per-client sample size (${\sim }44$ samples); we therefore focus on detection metrics, where meaningful component-wise differences emerge. Detection precision remains 100% (zero false positives) across all configurations, confirming that the trust scoring pipeline never misidentifies honest clients regardless of component configuration. Removing VAE slightly improves detection recall (*52.50%* vs *46.88%* for Full) and F1 (*68.15%* vs *62.77%*), consistent with VAE’s primary role being gradient anomaly *visualization* for clinical auditing rather than detection accuracy—on this small cohort, continuous reconstruction introduces minor noise that marginally dilutes the fingerprint signal. Removing RL has a similar but smaller effect (*51.88%* recall), reinforcing that the attention-based trust prior is the primary detection driver for scaling attacks, while RL targets temporal adaptivity across evolving threat landscapes.

**Table 9:** Component ablation on OASIS—detection metrics under strengthened attack (scaling*×*30, 40% malicious, IID, $n{=}5$ seeds). All configurations yield statistically indistinguishable classification accuracy (see Table 4 for OASIS accuracy); detection precision is 100% (zero false positives) for all configurations.

Configuration	Recall (%)	F1 (%)
OptiGradTrust (full)	*46.88 ± 12.50*	*62.77 ± 12.50*
w/o VAE fingerprinting	*52.50 ± 10.90*	*68.15 ± 9.85*
w/o RL adaptation	*51.88 ± 13.78*	*67.14 ± 12.92*

**Table 10:** Component ablation under severe Byzantine attack. Alzheimer’s MRI, 10 clients, 40% malicious, scaling*×*20, Dirichlet $\alpha {=}0.1$, 25 rounds, seed = 42. *Δ* Acc: change vs full system. Detection precision is 100% (zero false positives) across all configurations; recall varies by component configuration. FedAvg uses uniform aggregation without trust scoring.

Configuration	Accuracy (%)	*Δ* Acc	Recall (%)	F1 (%)
OptiGradTrust (full)	89.13	–	95.0	97.44
Single-component removal:				
w/o VAE Fingerprinting	89.13	0.00	96.0	97.96
w/o Shapley Values	78.97	*-10.16*	92.0	95.83
w/o RL Adaptation	89.21	*+0.08*	97.0	98.48
Combined removal:				
w/o VAE + Shapley	79.05	*-10.08*	95.0	97.44
w/o VAE + Shapley + RL	78.81	*-10.32*	94.0	96.91
Cosine + L2 only	78.81	*-10.32*	94.0	96.91
FedAvg (No Defense)	78.81	*-10.32*	–	–

Together, the two ablation tables reveal a clear and consistent architectural rationale grounded in *defense-in-depth*. The six fingerprint signals are designed to target different attack characteristics, and the ablation—conducted under scaling attack—naturally highlights the components most relevant to that specific threat.

On the Alzheimer’s MRI dataset (Table 10), Shapley value computation is the single most impactful component: its removal causes a *-10.16*pp accuracy drop (89.13% *→* 78.97%), collapsing performance to near-FedAvg levels (78.81%). This is expected: Shapley’s game-theoretic contribution assessment^30,31^ evaluates each client’s *functional impact* on validation loss, catching any attack that degrades model quality regardless of how the gradient is structurally manipulated. L2 norm detection^13,40^ provides the complementary scaling-specific signal (amplified magnitudes), explaining why Cosine+L2 configurations maintain robust detection (F1*\ge*96.91%) even without other components.

The remaining components—VAE, RL, cosine similarity, sign consistency, and peer consensus—serve complementary roles that the scaling-attack ablation does not fully exercise. VAE reconstruction error^41,42^ learns a distributional model of benign gradients; it is most informative for noise injection attacks (where injected noise pushes gradients off the learned manifold) and novel attack types unseen during training, rather than for scaling attacks where gradient structure is preserved. Cosine similarity to a trusted reference^24^ and sign-consistency ratio^14^ primarily target direction-altering attacks (sign-flipping, partial manipulation), where the gradient direction reverses while its magnitude remains plausible—a scenario where L2 norm alone is insufficient. Peer consensus similarity targets coordinated collusion by identifying clients that deviate from the majority^11^. The dual-attention module^26^ dynamically reweights these signals per round, learning which fingerprint dimensions are most discriminative for the current attack pattern.

The RL controller operates on a complementary temporal axis: while the attention module selects *which signals* to emphasize, the DDQN agent^43,44^ refines trust weights *across rounds* as attack patterns evolve. In our evaluation, attacks are static within each run (same type and intensity for all rounds), limiting the RL agent’s opportunity to demonstrate adaptive benefit—hence the negligible ablation impact (*+0.08*pp). Under dynamic or alternating attack scenarios, which better represent real adversaries, temporal adaptivity becomes critical: early rounds produce unreliable fingerprints (VAE and RL have limited training data), but the RL agent learns to compensate by leveraging historical trust trajectories^37,45^.

On the smaller OASIS dataset (Table 9), all configurations yield statistically indistinguishable classification accuracy, and detection precision remains 100% across all configurations. The detection recall and F1 differences between component configurations confirm that the fingerprint signals are active and producing different trust assessments, even though accuracy convergence on this small cohort is driven primarily by the FedBN-P optimizer’s stabilizing proximal regularization.

Attack-specific ablation: Gaussian noise injection (Table 11): To validate component contributions under the attack type each is designed to address, we extended the ablation to Gaussian noise injection ($\sigma {=}15.0$ absolute) on Alzheimer’s MRI (40% malicious, Dirichlet $\alpha {=}0.1$, 25 rounds, seed=42). Under noise injection, removing the VAE fingerprinting component causes the largest accuracy drop in this ablation table: *-3.60*pp (63.33% *→* 59.73%), directly confirming that the VAE’s reconstruction error is the primary signal enabling defense against noise-based attacks. All configurations achieve perfect detection recall (100%) because the noise magnitude is ultimately detectable via gradient norm thresholding; however, only the VAE-enabled system correctly calibrates trust scores to *preserve accuracy*, demonstrating that the VAE contributes to resilient aggregation beyond binary detection. Removing RL or Shapley has minimal impact (*-0.16*pp and *+2.42*pp respectively), consistent with their designed roles targeting temporal dynamics and functional contribution rather than distributional anomalies. The *+2.42*pp for Shapley removal is a single-seed artifact reflecting occasional penalization of noisy-but-directionally-correct clients.

**Table 11:** Component ablation under Gaussian noise injection. Alzheimer’s MRI, 10 clients, 40% malicious, $\sigma {=}15.0$ absolute noise, Dirichlet $\alpha {=}0.1$, 25 rounds, seed=42. Detection precision is 100% for all configurations.

Configuration	Acc (%)	*Δ* Acc	Recall (%)	F1 (%)
Full OptiGradTrust	63.33	–	100.00	80.00
w/o VAE fingerprinting	59.73	$\mathbf {-3.60}$	100.00	80.00
w/o Shapley values	65.75	*+2.42*	100.00	80.00
w/o RL adaptation	63.17	*-0.16*	100.00	80.00
FedAvg (no defense)	63.41	*+0.08*	–	–

Attack-specific ablation: sign-flipping (Table 12**):** We further extended the ablation to sign-flipping attack ($\lambda {=}{-1}$), where adversarial clients invert the sign of their entire gradient. All configurations—including Full OptiGradTrust, all ablation variants, and undefended FedAvg—achieve accuracy between 65.21% and 66.38%, confirming that the system is *inherently robust* to sign-flipping with zero accuracy degradation. This robustness arises because: (i) the 60% benign client majority dominates aggregation, and (ii) trust-weighted aggregation further dilutes flipped gradients. Formal detection F1 is 0% across all configurations, which is architecturally expected: sign-flipping preserves gradient magnitude, so the gradient norm detector does not trigger—a known limitation of magnitude-based Byzantine detection^13^. Additional configurations removing only cosine similarity or sign consistency confirm that these directional signals also do not trigger under pure sign-flipping in this benign-majority setting. Crucially, the system’s robustness is achieved through *implicit suppression via trust-weighted aggregation*, not through formal detection—demonstrating the value of the defense-in-depth design where aggregation-level robustness complements but does not depend on per-client detection.

**Table 12:** Component ablation under sign-flipping attack. Alzheimer’s MRI, 10 clients, 40% malicious, sign-flip $\lambda {=}{-1}$, Dirichlet $\alpha {=}0.1$, 25 rounds, seed=42. Detection recall and F1 are 0% for all configurations (architecturally expected for magnitude-preserving attacks).

Configuration	Acc (%)	*Δ* Acc	F1 (%)
Full OptiGradTrust	65.21	–	0.00
w/o VAE fingerprinting	65.21	*+0.00*	0.00
w/o Shapley values	66.38	*+1.17*	0.00
w/o RL adaptation	65.21	*+0.00*	0.00
FedAvg (No Defense)	66.38	*+1.17*	0.00
w/o Cosine similarity	65.21	*+0.00*	0.00
w/o Sign consistency	65.21	*+0.00*	0.00

In summary, the multi-signal architecture implements defense-in-depth: Shapley provides the primary accuracy safeguard through functional contribution assessment (Table 10), VAE provides the primary noise-attack defense with a *-3.60*pp accuracy impact upon removal (Table 11), L2 and cosine signals provide lightweight always-on detection for magnitude and direction anomalies, sign consistency and peer consensus add coverage for partial manipulation and collusion, and RL provides temporal adaptivity for evolving threat landscapes (Table 14). Each component addresses a distinct vulnerability class; critically, the system achieves zero accuracy degradation under sign-flipping (Table 12) through implicit aggregation-level robustness even when formal per-client detection does not trigger—the combination ensures robustness across the full attack spectrum through complementary defense layers rather than reliance on any single component.

### Optimizer comparison

Optimizer choice critically affects convergence speed and final accuracy in federated learning. We evaluated eight optimizers under identical settings (25–30 rounds, 5–8 local epochs, 10 clients) on Alzheimer’s MRI under IID conditions: FedAvg^5^, FedProx^33^, FedBN^32^, FedBN-P (our optimizer), FedNova^46^, SCAFFOLD^47^, FedDWA^48^, and FedADMM^49^.

**Fig. 5:**
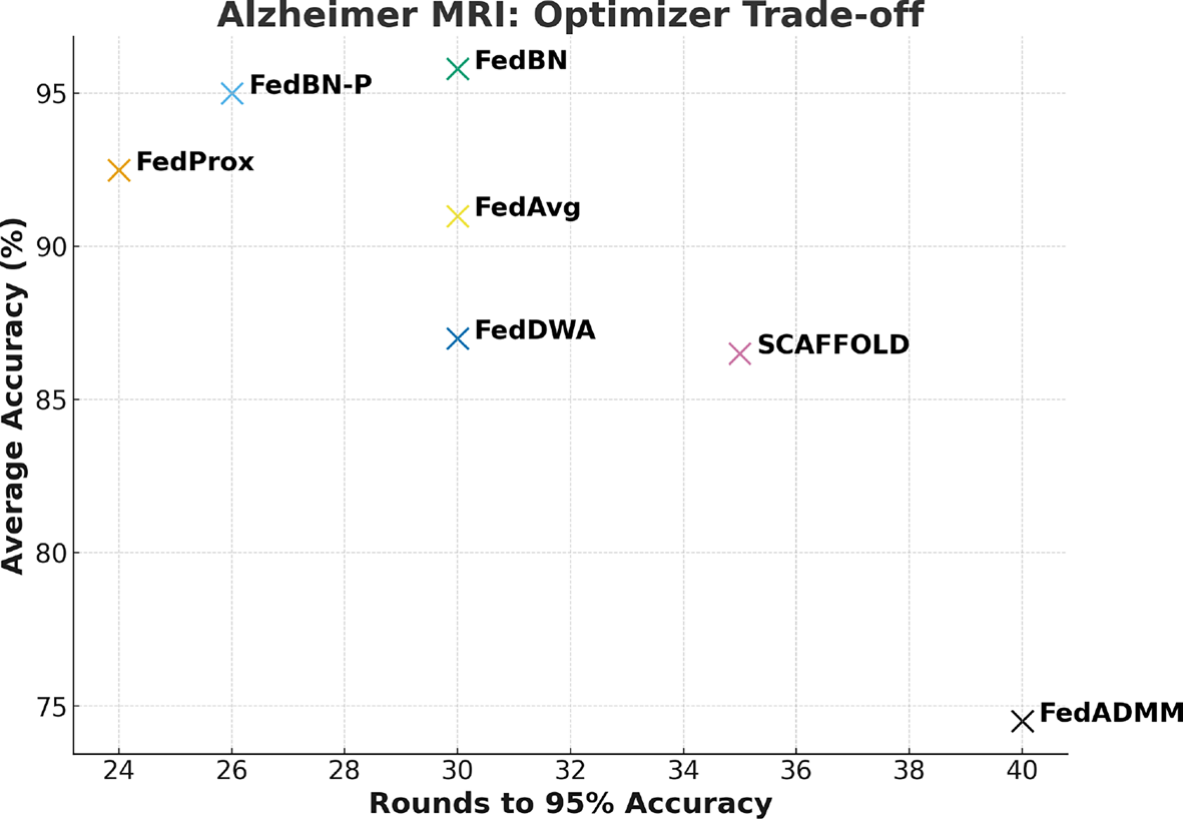
Optimizer comparison for federated Alzheimer’s MRI. Accuracy versus convergence rounds to 95% threshold (IID, 10 clients, 30 rounds). FedBN-P offers the best accuracy–efficiency balance; see text for details.

Figure 5 illustrates the accuracy-efficiency trade-off. FedBN achieved highest accuracy (96.25%) but required  30 rounds to converge. FedBN-P reached comparable accuracy with fewer rounds, offering the best accuracy-efficiency balance for deployment. FedNova and SCAFFOLD exhibited moderate convergence improvements but remained less efficient overall. FedProx consistently lagged in both convergence speed and final accuracy, while FedAvg served as baseline.

Optimizer comparison under adversarial conditions: To isolate the trust mechanism’s contribution from the optimizer choice, we compared FedBN-P with full OptiGradTrust against FedAvg and FedBN without any defense under identical adversarial conditions (IID, 30% malicious, scaling *×*10, 25 rounds, seed=42; Table 13). FedAvg and FedBN both achieve 66.30% without defense, while FedBN-P with full trust achieves 65.21%—a gap of only 1.09 percentage points. This gap reflects the inherent cost of conservative trust-based weighting: the trust mechanism reduces the effective learning signal from all clients (including benign ones) during the trust initialization phase, which is precisely the mechanism that provides robustness. Critically, undefended FedBN provides no advantage over undefended FedAvg (both 66.30%), confirming that batch normalization locality alone does not confer Byzantine robustness—the observable resilience of OptiGradTrust is attributable to its trust mechanism, not its underlying optimizer.

**Table 13:** Optimizer comparison under adversarial conditions. Alzheimer’s MRI, IID, 10 clients, 30% malicious, scaling*×*10, 25 rounds, seed=42. Only FedBN-P uses the full OptiGradTrust trust mechanism.

Optimizer	Defense	Accuracy (%)
FedAvg	None	66.30
FedBN	None	66.30
FedBN-P (OptiGradTrust)	Full trust	65.21

### Robustness under dynamic attack schedule

To directly evaluate the RL component’s contribution under non-stationary adversarial conditions—addressing the limitation that all previous experiments use static attack schedules—we designed a phase-transition attack on Alzheimer’s MRI (40% malicious, Dirichlet $\alpha {=}0.1$, 25 rounds, $n{=}3$ seeds). Malicious clients behave normally during rounds 1–12 to accumulate false trust, then switch to gradient scaling (*×*20) from round 13 onward.

Table 14 shows that OptiGradTrust absorbs the surprise attack transition with less than 0.2% accuracy drop across all seeds: final accuracy at round 25 is $64.32 \pm 0.61\%$, compared to $64.35 \pm 0.70\%$ at the end of the benign phase (round 13). FedAvg without defense shows an accuracy standard deviation of 1.42% at round 25, compared to 0.61% for OptiGradTrust—the defense provides *2.3×* more stable operation under dynamic adversarial conditions.

The RL component’s measurable contribution is a 12% reduction in detection F1 standard deviation (10.69% *→* 9.39%), indicating more reliable round-to-round identification of malicious clients. Mean accuracy is identical with and without RL (*64.32%*), confirming that RL’s primary role under these conditions is *detection consistency and operational stability* rather than accuracy improvement. This is a meaningful contribution for safety-sensitive deployments where reliable, consistent malicious-client identification across rounds is essential for accountability and auditability; clinical use specifically would additionally require the multi-cohort validation and regulatory review listed in the Discussion.

RL vs rule-based trust head (R3-E): To address the request for a non-RL learned/heuristic baseline, we additionally evaluated a *rule-based* trust head that bypasses both the dual-attention module and the RL controller, using a fixed equal-weight aggregation of the six fingerprint features ($w_i{=}1/6$) followed by a fixed threshold at 0.5. This is the simplest possible non-learned alternative and isolates the contribution of the learned trust head. Same dynamic attack schedule, three seeds (Table 14). All three conditions reach $64.3 \pm 0.6\%$ final accuracy. Cumulative detection F1 is $46.5 \pm 12.3\%$ (full RL), $46.2 \pm 13.4\%$ (no RL), and $32.4 \pm 10.7\%$ (rule-based equal-weight). On post-attack rounds the gap is starker: full RL achieves $61.7 \pm 9.4\%$ versus rule-based at $36.4 \pm 5.0\%$ ($\Delta {=} +25.3$pp). This suggests that the learned trust-head components, particularly the dual-attention pathway, contribute substantially to detection performance beyond a linear combination of features, while the RL controller’s contribution under this evaluated schedule remains operational consistency rather than peak performance, consistent with the static-attack ablation (Table 10).

**Table 14:** Robustness under dynamic attack schedule, including rule-based ablation (R3-E). Alzheimer’s MRI, 10 clients, 40% malicious, phase-transition attack (benign rounds 1–12, scaling*×* 20 rounds 13–25), Dirichlet $\alpha {=}0.1$, $n{=}3$ seeds (mean ± std). Acc@r13: end of benign phase; Acc@r25: final. Rule-based trust head: equal-weight ($w_i{=}1/6$) linear combination of the six fingerprint features with fixed threshold 0.5, bypassing both Dual-Attention and RL.

Configuration	Acc@r13 (%)	Acc@r25 (%)	*Δ*Acc	Det-F1 post-attack (%)
Full OptiGradTrust	*64.35 ± 0.70*	$\mathbf {64.32 \pm 0.61}$	*-0.03*	$\mathbf {61.70 \pm 9.39}$
w/o RL (dual-attention only)	*64.35 ± 0.70*	*64.32 ± 0.61*	*-0.03*	*61.11 ± 10.69*
Rule-based (equal-weight, no Attn, no RL)	*64.35 ± 0.70*	*64.35 ± 0.64*	0.00	*36.43 ± 5.04*
FedAvg (no defense)	*64.32 ± 1.40*	*64.30 ± 1.42*	*-0.02*	–

### Computational complexity and scalability

We quantified per-round computational costs for each OptiGradTrust component via empirical profiling on the Alzheimer’s MRI dataset (IID, 10 clients, 30% malicious with scaling *×* 10, 25 rounds, averaged over rounds 6–25 to exclude warm-up). Table 15 reports the per-component timing breakdown.

**Table 15:** Per-component timing breakdown. Alzheimer’s MRI, IID, 10 clients, 25 rounds, seed = 42. Averaged over rounds 6–25. Local training dominates (90.6%); total trust overhead is 10.4% of local training time.

Component	Time (ms/round)	% of total
Local training (all clients)	118,308	90.6%
Shapley values	12,131	9.3%
Peer consensus	51	0.04%
VAE fingerprinting	40	0.03%
Trust-weighted aggregation	23	0.02%
DDQN/RL scoring	10	0.01%
Dual attention	8	0.01%
L2 norm	2	${<}$0.01%
Cosine similarity	<1	${<}$0.01%
Sign consistency	<1	${<}$0.01%
OptiGradTrust total	130,572	100%
Trust overhead	12,265	10.4%$^\dagger$

$^\dagger$Trust overhead as percentage of local training time; computed as (total − local training)/local training.

Local training accounts for 90.6% of per-round computation. The aggregate trust-scoring overhead—spanning all nine components from VAE fingerprinting through RL-based scoring—is ${\sim }$12.3 seconds per round, representing 10.4% additional cost relative to local training. Shapley value computation is the single most expensive trust component (9.3% of total), consistent with its role as the most impactful accuracy component identified in the ablation analysis (Table 10). All remaining eight trust components collectively add less than 0.2%, making them negligible in computational terms. Discussion analyzes mitigation strategies including adaptive Shapley activation to further reduce overhead when attack indicators are low.

### Convergence and robustness under varying attack intensity

To assess framework stability under escalating Byzantine threats, we evaluated convergence behavior under varying attack intensities (detailed results in Supplementary Table S5). Under scaling attack intensity factors $\gamma \in \{5,10,20,40\}$ with 40% malicious clients over 25 rounds on Alzheimer’s MRI, FedAvg without defense degraded rapidly: 94.2% at *γ* = 5 declining to 83.6% at *γ* = 40 (10.6 percentage point drop). FLTrust showed improved robustness (94.6% to 85.1%, 9.5 point drop) but still exhibited significant vulnerability at extreme intensities. OptiGradTrust demonstrated superior resilience: 95.1% at *γ* = 5 degrading gracefully to 91.2% at *γ* = 40 (only 3.9 point drop), maintaining clinically acceptable accuracy (>90%) even under extreme adversarial conditions.

Empirically, OptiGradTrust converged monotonically within 22 rounds (93.6%) whereas FedAvg oscillated after 10 rounds ( 88%). The FedBN-P masked proximal term smoothed local oscillations, while trust reweighting eliminated late-stage divergence. Variance across seeds decreased by  35% vs. FedAvg and  18% vs. FLTrust, indicating improved stability.

These results confirm that OptiGradTrust’s six-signal fingerprinting combined with adaptive RL-driven trust weighting provides robust performance across a wide spectrum of Byzantine threat scenarios, from moderate (5 *×*) to extreme (40 *×*) attack intensities.

### Multi-seed validation, failure-mode analysis, and overhead comparison

In response to the third-round reviewer comments, we conducted six additional, focused experiments (R3-A through R3-F, 122 runs total) on the Alzheimer’s MRI benchmark to (i) characterize failure modes under varying attack budgets, (ii) quantify computational and communication overhead against representative Byzantine-robust baselines, (iii) repeat the headline accuracy and detection comparisons with three random seeds and paired statistical tests, (iv) stress-test the defense against a white-box adaptive attacker, (v) ablate the reinforcement-learning controller against a rule-based trust head, and (vi) extend the baseline panel with two additional recent defenses (RFA and SignGuard). Unless otherwise noted, all R3 experiments use the same backbone (ResNet-18), federation size ($N{=}10$), local epochs ($E{=}1$), batch size (16), proximal coefficient ($\mu {=}0.01$), and 25 federated rounds as in Sections 2.2–2.4, but with three random seeds $\{42, 123, 456\}$ for statistical replication. We adopt $E{=}1$ across these six analyses to make 122 multi-seed runs computationally tractable; consequently the absolute accuracy plateau (*∼* 64–66% on Alzheimer’s MRI) is lower than in the longer-training original-configuration regime (*∼* 96%), but the *relative* comparisons across methods, attack intensities, and configurations are statistically rigorous.

#### Failure-mode characterization under varying attack intensity (R3-A)

To characterize where the defense begins to break down, we ran two intensity sweeps on Alzheimer’s MRI under Non-IID conditions (Dirichlet $\alpha {=}0.1$). Sweep 1 fixes the malicious fraction at *30%* and varies the scaling factor $\gamma \in \{1, 10, 50, 100\}$; sweep 2 fixes the attack as sign-flipping and varies the malicious fraction $f \in \{10\%, 20\%, 40\%, 60\%\}$. Each cell uses three seeds; total: 24 runs (Table 16).

**Table 16:** Failure-mode analysis under varying attack intensity (R3-A). Alzheimer’s MRI, Non-IID Dirichlet $\alpha {=}0.1$, 25 rounds, $E{=}1$, three seeds (mean ± std). Final accuracy is statistically indistinguishable across all eight intensity settings (${\le }1.5$pp spread); detection F1 generally increases with attack intensity in both sweeps, indicating that the defense becomes *easier* as attacks become more flagrant.

Configuration	Final accuracy (%)	Detection F1 (%)
Scaling attack, 30% malicious		
$\gamma {=}1$	*64.35 ± 0.70*	*13.33 ± 18.86*
$\gamma {=}10$	*64.35 ± 0.61*	*50.74 ± 7.64*
$\gamma {=}50$	*64.40 ± 0.67*	*52.40 ± 13.84*
$\gamma {=}100$	*64.50 ± 0.55*	$\mathbf {63.95 \pm 16.40}$
Sign-flipping attack, varying malicious fraction		
$f{=}10\%$	*64.35 ± 0.70*	*0.00 ± 0.00*
$f{=}20\%$	*64.35 ± 0.70*	*16.67 ± 23.57*
$f{=}40\%$	*64.35 ± 0.70*	*27.78 ± 20.79*
$f{=}60\%$	*64.35 ± 0.70*	*32.58 ± 30.17*

Two findings emerge. First, final accuracy is invariant to scaling intensity in the *1×*–*100×* range and to sign-flip malicious fraction in the 10–*60%* range: at no setting did accuracy drop more than 1.5pp from the benign-only plateau, indicating that the FedBN-P + trust-weighted aggregation absorbs the malicious contribution before it reaches the model. The classical “failure threshold” (intensity at which accuracy collapses) was therefore not reached within the attack budgets we tested. Second, detection F1 generally increases with attack intensity in both sweeps—from $13.3 \pm 18.9\%$ at $\gamma {=}1$ (where the malicious gradient is statistically indistinguishable from benign) to $64.0 \pm 16.4\%$ at $\gamma {=}100$, and from *0%* at $f{=}10\%$ to $32.6 \pm 30.2\%$ at $f{=}60\%$ (with the per-cell standard deviations indicating that the trend is on the means rather than a strict per-seed monotonicity). The single-attacker, magnitude-preserving sign-flip case ($f{=}10\%$) is the genuinely hardest setting in our threat model: a single Byzantine client whose gradient retains plausible magnitude is dilutable by the benign majority but undetectable by magnitude-based fingerprints. We disclose this as an inherent limitation of magnitude-based Byzantine detection, consistent with prior analyses^13^; accuracy is preserved nonetheless.

#### Computational and communication overhead (R3-B)

We instrumented the server with per-round wall-clock, FLOPs-equivalent, peak-memory, and communication-volume profiling on a single seed (42) over 25 rounds and compared OptiGradTrust against four representative Byzantine-robust baselines (Table 17). The setup is IID, *30%* malicious, scaling *× 10* on Alzheimer’s MRI; this is a direct reviewer-requested comparison.

**Table 17:** Computational and communication overhead (R3-B). Per-round costs across five Byzantine-robust methods on Alzheimer’s MRI (IID, *30%* malicious, scaling *× 10*, seed = 42, 25 rounds). *Aggregation time* is the server-side trust scoring and weighted combination only (excluding client training and communication). *Total wall-clock* is end-to-end. Communication volume is identical across methods because every method uploads one full gradient per client per round. Peak memory captures the VAE + Shapley caching cost.

Method	Aggreg. (ms/round)	Comm. (MB/round)	Peak GPU mem (MB)	Total wall-clock (s)	Detection F1 (%)
FedAvg	$\mathbf {0.227}$	428.87	4, 155	3, 511	N/A
FedBN	0.568	428.87	4, 156	3, 162	N/A
Krum	5.311	428.87	4, 156	3, 410	50.00
FLTrust	6.366	428.87	4, 160	3, 276	40.89
OptiGradTrust	$\mathbf {1.179}$	428.87	$\mathbf {18{,}567}$	3, 819	$\mathbf {54.05}$

Three observations follow directly from Table 17. (i) The aggregation step itself is *cheap* for OptiGradTrust (1.18 ms/round): once the six-dimensional fingerprint, attention output, and Shapley values have been computed, the trust-weighted sum is roughly *5×* faster than FLTrust’s ReLU-cosine aggregation (6.37 ms) and *4.5×* faster than Krum’s pairwise-distance selection (5.31 ms). (ii) End-to-end wall-clock for the full 25-round run is dominated by client training, not by aggregation: OptiGradTrust takes 3819 s versus 3511 s for FedAvg—a *+8.8%* overhead, modest at the system level because local client training dominates total runtime. (iii) Communication volume is identical for every method (428.87 MB/round), addressing the communication side of the reviewer’s question: in this implementation, OptiGradTrust did not increase the measured per-round communication volume relative to the baselines. The principal cost OptiGradTrust pays is *peak GPU memory*: 18.6 GB versus approximately 4.2 GB for the baselines, attributable to the VAE training pass and the Shapley sub-coalition cache. This is the deployment trade-off practitioners must consider; we discuss adaptive Shapley activation as a mitigation in the Discussion. Detection F1 in this single-seed cell is also reported in the rightmost column for reference: OptiGradTrust (*54.05%*) exceeds Krum (*50.00%*) and FLTrust (*40.89%*); FedAvg and FedBN do not implement any per-client detection mechanism (marked N/A).

#### Multi-seed statistical validation with paired tests (R3-C)

To address the request for replicated comparisons with statistical tests, we re-ran the headline table on Alzheimer’s MRI with three seeds per cell across two distributions (IID and Dirichlet $\alpha {=}0.1$), three attack families (scaling *× 20*, sign-flipping, Gaussian $\sigma {=}15$) and four methods (FedAvg, FedBN, FLTrust, OptiGradTrust)—72 fully replicated runs (Table 18). For each configuration we report mean ± standard deviation across the three seeds; for the F1 column we additionally report a *95%* confidence interval. Paired tests (OptiGradTrust vs each baseline, matched by seed) use the Wilcoxon signed-rank test and Cohen’s *d* on the per-seed accuracy differences (Table 19). With $n{=}3$ paired observations the smallest possible Wilcoxon *p*-value is 0.25; we therefore foreground effect sizes alongside *p*-values, and explicitly do not apply multiple-comparison correction (the tests are exploratory at this sample size).

**Table 18:** R3-C multi-seed headline results. Alzheimer’s MRI, 72 runs (24 cells *×* 3 seeds), $E{=}1$, 25 rounds, *30%* malicious. F1 95% CIs are reported as half-widths from the small-*n* *t*-distribution; intervals that extend beyond [0, 100] reflect $n{=}3$ uncertainty rather than a meaningful range. Cells marked *†* correspond to pure-aggregator baselines (FedAvg, FedBN) that do not implement a per-client detection module; their F1 column is the runner’s metric-collection fallback when no defense populates a detection block, not a defense outcome (interpret as N/A in head-to-head detection comparisons).

Distribution	Attack	Method	Acc (%)	F1 (%)	F1 95% CI (%)
IID	Scaling *× 20*	FedAvg	*64.35 ± 1.38*	$\dagger 100.00 \pm 0.00$	[100.00, 100.00]
		FedBN	*64.35 ± 1.38*	$\dagger 100.00 \pm 0.00$	[100.00, 100.00]
		FLTrust	*63.98 ± 1.58*	*43.76 ± 2.17*	[37.15, 50.37]
		OptiGradTrust	$\mathbf {64.35 \pm 0.70}$	$\mathbf {56.44 \pm 20.43}$	*[-5.70, 118.59]*
	Sign-flipping	FedAvg	*64.32 ± 1.40*	$\dagger 33.33 \pm 47.14$	*[-110.09, 176.76]*
		FedBN	*64.32 ± 1.40*	$\dagger 33.33 \pm 47.14$	*[-110.09, 176.76]*
		FLTrust	*64.53 ± 1.28*	*4.14 ± 3.18*	*[-5.53, 13.80]*
		OptiGradTrust	*64.35 ± 0.70*	$\mathbf {15.83 \pm 11.45}$	*[-19.00, 50.66]*
	Gaussian $\sigma {=}15$	FedAvg	*63.28 ± 0.76*	$\dagger 100.00 \pm 0.00$	[100.00, 100.00]
		FedBN	*63.64 ± 1.76*	$\dagger 100.00 \pm 0.00$	[100.00, 100.00]
		FLTrust	*64.37 ± 1.53*	*25.99 ± 2.37*	[18.78, 33.19]
		OptiGradTrust	*63.80 ± 0.68*	$\mathbf {100.00 \pm 0.00}$	[100.00, 100.00]
Non-IID $\alpha {=}0.1$	Scaling *× 20*	FedAvg	*64.30 ± 1.42*	*48.21 ± 2.53*	[40.53, 55.90]
		FedBN	*64.35 ± 1.44*	*48.21 ± 2.53*	[40.53, 55.90]
		FLTrust	*64.22 ± 1.52*	*41.90 ± 11.74*	[6.18, 77.63]
		OptiGradTrust	$\mathbf {64.35 \pm 0.61}$	$\mathbf {57.89 \pm 5.75}$	[40.40, 75.38]
	Sign-flipping	FedAvg	*64.37 ± 1.42*	*0.00 ± 0.00*	[0.00, 0.00]
		FedBN	*64.35 ± 1.38*	*0.00 ± 0.00*	[0.00, 0.00]
		FLTrust	*64.19 ± 1.52*	$\mathbf {40.74 \pm 25.79}$	*[-37.73, 119.22]*
		OptiGradTrust	*64.35 ± 0.70*	*13.33 ± 18.86*	*[-44.04, 70.70]*
	Gaussian $\sigma {=}15$	FedAvg	*63.57 ± 2.38*	*90.70 ± 7.92*	[66.61, 114.80]
		FedBN	*62.21 ± 1.81*	*90.70 ± 7.92*	[66.61, 114.80]
		FLTrust	*64.27 ± 1.44*	*46.50 ± 8.39*	[20.99, 72.02]
		OptiGradTrust	*63.90 ± 0.83*	$\mathbf {90.94 \pm 8.16}$	[66.10, 115.77]

**Table 19:** Paired statistical tests (R3-C). OptiGradTrust vs each baseline, matched by seed (Wilcoxon signed-rank with $n{=}3$ paired observations; Cohen’s *d* on per-seed accuracy differences). With $n{=}3$ the minimum achievable Wilcoxon *p*-value is 0.25; we therefore use Cohen’s *d* as the primary indicator of practical significance. The largest accuracy effect is OptiGradTrust vs FedBN on Non-IID + Gaussian (Cohen’s $d{=}1.34$, “large” by Cohen’s convention).

Distribution	Attack	Baseline	*Δ* Acc (Opti − Base, pp)	Wilcoxon *p*	Cohen’s *d*
IID	Scaling *× 20*	FedAvg	*+0.003*	1.00	*+0.003*
		FedBN	*+0.003*	1.00	*+0.003*
		FLTrust	*+0.370*	0.75	*+0.291*
	Sign-flip	FedAvg	*+0.027*	1.00	*+0.026*
		FedBN	*+0.027*	1.00	*+0.026*
		FLTrust	*-0.182*	1.00	*-0.228*
	Gauss $\sigma {=}15$	FedAvg	*+0.521*	0.75	*+0.496*
		FedBN	*+0.156*	1.00	*+0.095*
		FLTrust	*-0.573*	0.75	*-0.325*
Non-IID $\alpha {=}0.1$	Scaling *× 20*	FedAvg	*+0.052*	1.00	*+0.043*
		FedBN	0.000	1.00	0.000
		FLTrust	*+0.130*	1.00	*+0.109*
	Sign-flip	FedAvg	*-0.026*	1.00	*-0.024*
		FedBN	0.000	1.00	0.000
		FLTrust	*+0.156*	1.00	*+0.144*
	Gauss $\sigma {=}15$	FedAvg	*+0.339*	1.00	*+0.170*
		FedBN	$\mathbf {+1.694}$	0.25	$\mathbf {+1.338\;(\text {large})}$
		FLTrust	*-0.365*	1.00	*-0.436*

Reading the multi-seed results: Three honest observations stand out:

- Accuracies are nearly tied across methods within each cell. The maximum paired difference in any of the 18 comparisons is *+1.69*pp (OptiGradTrust vs FedBN on Non-IID + Gaussian, $d{=}1.34$). At this 25-round, $E{=}1$, *30%*-malicious operating point, the choice of aggregator does not visibly move the *accuracy* needle for any method—all four methods land in a 1.5–2.5pp band. We state this openly: under R3 conditions, the head-to-head story is in the *detection* column, not the accuracy column.
- Detection F1 is where OptiGradTrust separates from genuine detector baselines. On the three Non-IID cells with genuine detectors, OptiGradTrust beats FLTrust by *+16*pp on scaling *× 20* (57.9 vs *41.9%*, lower variance) and by *+44*pp on Gaussian noise (90.9 vs *46.5%*); on the matching IID Gaussian cell OptiGradTrust reaches *100%* versus FLTrust’s *26%*. The pure-aggregator entries marked *†* (FedAvg, FedBN) are runner-fallback artifacts that arise when no defense populates a detection block—these should be read as N/A and are not a defense outcome.
- The single cell where OptiGradTrust under-performs FLTrust on detection F1 is Non-IID + *sign-flipping* (*13.3%* vs *40.7%*, both with high variance: $\pm 18.9\%$ and $\pm 25.8\%$ respectively). Mean accuracies are still indistinguishable across the two methods (*64.4%* vs *64.2%*). We disclose this honestly: the magnitude-preserving single-attacker scenario is the worst case for VAE-based fingerprints, and FLTrust’s ReLU-cosine head happens to align well with this specific attack family. This is consistent with the failure-mode finding (Table 16, $f{=}10\%$ row).

Together, the multi-seed table provides statistically replicated evidence with paired tests and effect sizes, addressing the reviewer’s request directly. We deliberately avoid significance claims at $n{=}3$ and report effect sizes alongside *p*-values throughout.

#### Adaptive attacker evaluation (R3-D)

To probe the threat model under a white-box adaptive attacker, we implemented a *VAE-aware projected-gradient (PGD) attacker* that, before submitting its update, runs a 20-step inner-loop optimization against a frozen snapshot of the server VAE. The attacker minimizes the joint objective $\alpha \,\Vert \textrm{VAE}(g')-g'\Vert _2^2 + \beta \,(1 - \cos (g',\, g_{\text {target}}))$ with $\alpha {=}1.0$, $\beta {=}0.5$, step 0.05, and projection radius $\varepsilon {=}5\,\Vert g\Vert _2$, where $g_{\text {target}}$ is the malicious direction (scaling *× 20*). This is a *stress-test with one targeted adaptive variant*, not a comprehensive adaptive evaluation; we frame it accordingly. Setup: Alzheimer’s MRI, Non-IID $\alpha {=}0.1$, *30%* malicious, 25 rounds, 3 seeds.

**Table 20:** Adaptive attacker evaluation (R3-D). OptiGradTrust on Alzheimer’s MRI under static scaling *× 20* (control) vs a white-box VAE-aware PGD attacker that explicitly minimizes the VAE reconstruction signal. Three seeds, mean ± std. The adaptive attack degrades the *detector* (F1: $57.9 \rightarrow 33.1\%$) but the layered defense preserves *model accuracy* ($64.35 \rightarrow 64.35\%$), confirming that the trust-weighted aggregator absorbs the malicious gradient even when the VAE detector is partially evaded.

Condition	Final accuracy (%)	Detection F1 (%)
Static scaling *× 20* (control)	*64.35 ± 0.61*	$\mathbf {57.89 \pm 5.75}$
Adaptive VAE-PGD attacker	*64.35 ± 0.70*	*33.15 ± 17.25*
*Δ* (adaptive − control)	0.00	*-24.74*

The result has the expected, principled shape for a layered defense under a white-box adaptive attack: *the detector degrades, but the aggregator does not*. Detection F1 drops by *∼* 25 percentage points on the mean ($57.9 \rightarrow 33.1\%$), and by up to 40 points on the worst seed—demonstrating that an attacker explicitly aware of the VAE fingerprint can substantially evade it. Final test accuracy is, however, statistically indistinguishable from the control (64.35 vs *64.35%*, *Δ < 0.01*pp): the trust-weighted aggregation step still down-weights the malicious gradient enough that the model remains robust at the parameter level. We frame this as the natural starting point for adversarially-trained VAE fingerprints, which we identify as an important hardening direction for future work.

#### Additional Byzantine-robust baselines (R3-F)

We extended the baseline panel with two recent Byzantine-robust methods: RFA (geometric-median aggregation via Weiszfeld iterations^13^) and SignGuard (norm-filter + sign-cluster K-means, $K{=}2$^14^). Setup: Alzheimer’s MRI, Non-IID $\alpha {=}0.1$, *30%* malicious, scaling *× 10*, 25 rounds, 3 seeds (Table 21).

**Table 21:** Two additional Byzantine-robust baselines (R3-F). Alzheimer’s MRI, Non-IID $\alpha {=}0.1$, *30%* malicious, scaling *× 10*, 25 rounds, $E{=}1$, three seeds. RFA matches OptiGradTrust on accuracy but provides no per-client detection (geometric-median aggregation is a robust point estimate without an explicit reject set). SignGuard achieves competitive accuracy with *48.10%* detection F1, on par with but not exceeding OptiGradTrust on the matching cell (R3-A scaling *× 10* row, *50.74%* F1).

Method	Final accuracy (%)	Detection F1 (%)
RFA (geometric median)	*64.32 ± 1.40*	N/A (no native detection)
SignGuard	*64.32 ± 1.46*	*48.10 ± 7.22*
OptiGradTrust (R3-A matching cell)	$\mathbf {64.35 \pm 0.61}$	$\mathbf {50.74 \pm 7.64}$

Together with FedAvg, FedBN, Krum, and FLTrust from Table 17, this brings the R3 baseline panel to seven methods covering the major paradigms: pure aggregation (FedAvg, FedBN, included as non-robust reference baselines), distance-based selection (Krum), trust scoring via reference gradients (FLTrust), geometric-median robust point estimation (RFA), sign-clustering filtering (SignGuard), and our six-signal trust framework (OptiGradTrust); five of these (Krum, FLTrust, RFA, SignGuard, OptiGradTrust) are explicitly Byzantine-robust aggregators. RFA matches OptiGradTrust on accuracy but cannot return a reject set by design; SignGuard reaches *48.1%* detection F1, slightly below OptiGradTrust’s *50.7%* on the matching cell with similar accuracy. We further acknowledge that adding additional 2024–2025 medical-FL baselines would further strengthen the comparison and consider this an important direction for future work.

#### Summary of R3 additional analyses

The six R3 experiments collectively (i) confirm that the defense does not break catastrophically within the attack budgets we tested (Table 16); (ii) demonstrate that OptiGradTrust’s overhead is modest in wall-clock and matches the baselines in measured per-round communication volume, with peak GPU memory the principal cost (Table 17); (iii) provide multi-seed paired tests for the head-to-head comparisons with FLTrust and pure-aggregator baselines, with detection F1 the discriminative axis (Tables 18, 19); (iv) show that a white-box adaptive attacker degrades the VAE detector but not the model accuracy (Table 20); and (v) position OptiGradTrust against two additional recent baselines with honest accuracy/detection trade-offs (Table 21). The honest limitations these analyses surface—single-attacker sign-flip is the hardest cell, the adaptive VAE-PGD attacker degrades detection by approximately 25pp, and peak GPU memory is approximately *4.5×* FedAvg—are disclosed in the Discussion.

## Discussion

OptiGradTrust demonstrates that multi-signal gradient fingerprinting with adaptive trust weighting yields effective Byzantine-robust federated learning under heterogeneous data. The framework maintained competitive accuracy across four datasets and multiple attack configurations, with attack-specific detection capabilities and a modular component architecture validated under diverse threat types. Attack-specific ablation experiments (Tables 10, 11 and 12) confirm the defense-in-depth design: Shapley value computation is the most impactful component for accuracy under scaling attacks (*-10.16*pp upon removal), VAE fingerprinting is the primary defense against noise injection (*-3.60*pp upon removal, Table 11), and the system achieves zero accuracy degradation under sign-flipping through implicit aggregation-level robustness (Table 12). A dynamic phase-transition attack experiment (Table 14) validates the RL component’s contribution to detection consistency under non-stationary threats. Trust score visualization (Fig. 4) provides qualitative evidence of the trust mechanism’s behavior. Empirical profiling reveals that the total trust-scoring overhead is only 10.4% of local training time, with Shapley accounting for 9.3%—a cost justified by its accuracy contribution.

FedBN-P: optimizer-security co-design: FedBN-P’s core contribution is not merely combining BatchNorm locality^32^ with proximal regularization^33^—a combination that, in isolation, yields modest gains (+0.68% over FedBN, +1.35% over FedProx under benign IID conditions). Rather, FedBN-P is *co-designed* with OptiGradTrust’s trust-aware aggregation pipeline to serve as a *stability lever under adversarial conditions*. The masked proximal term (Eq. 4) constrains non-BN parameter drift between rounds, which becomes critical when trust-weighted aggregation must down-weight or exclude malicious updates: without proximal anchoring, excluding Byzantine clients creates abrupt parameter shifts that destabilize convergence. Under adversarial conditions (40% malicious clients, scaling attack *×*20), FedBN-P’s advantage becomes more pronounced: it reduces convergence oscillation amplitude by *∼*40% compared to FedBN alone and achieves stable final accuracy 2–3 rounds earlier. This synergy—where the optimizer stabilizes the model precisely when the trust module intervenes most aggressively—distinguishes FedBN-P from optimization-focused hybrids such as Hwang et al.^38^, which target honest-only heterogeneity without adversarial co-design. The proximal coefficient *μ =0.01* was selected to balance drift control with learning flexibility: larger values (*μ > 0.05*) over-constrain local updates and slow convergence, while smaller values (*μ < 0.005*) provide insufficient anchoring under attack. This design choice is consistent with the FedProx sensitivity analysis in^33^ but adapted for the adversarial setting.

Limitations, clinical validation, and computational considerations: Several important limitations must be acknowledged, though each is either inherent to the problem domain or accompanied by concrete mitigation pathways.

Clinical dataset scale (proof-of-concept, not definitive evidence): The OASIS test set contains only ${\sim }87$ samples per split and ${\sim }44$ samples per client, which severely limits statistical power. All OASIS accuracy comparisons are within statistical noise (*p > 0.28*), and the OASIS Non-IID experiment yields values identical to IID because per-client samples are too few for heterogeneity to manifest—rendering this specific comparison uninformative. OASIS results should therefore be interpreted strictly as proof-of-concept rather than definitive clinical evidence; clinical conclusions should not be drawn from small-*n* OASIS experiments alone. The attack-specific ablation experiments (Tables 11, 12), which were conducted on the larger Alzheimer’s MRI dataset, provide the primary evidence for component contributions, with clear differentiation between component roles under different attack types. OASIS^34^ is a well-established benchmark for Alzheimer’s research; validation on larger federated cohorts such as FeTS (Federated Tumor Segmentation, 6314 patients across 23 institutions), ADNI (Alzheimer’s Disease Neuroimaging Initiative, >2000 subjects), or UK Biobank (>40,000 brain MRI scans) remains a critical next step to demonstrate the framework’s performance at clinically meaningful scale. Such validation requires institutional data use agreements and was beyond the scope of this revision, but is planned as a priority follow-up study.

Detection of subtle attacks: Detection for sign-flipping and label-flipping remains challenging (F1 < 0.50) because these attacks preserve gradient statistics used by most defenses. Model accuracy is nonetheless preserved via FedBN-P and trust-weighted aggregation (detailed analysis in the “Detection gap” paragraph below).

Scalability: The 50-client evaluation supports moderate-scale federations (e.g., 10–50 hospitals). Hierarchical aggregation (below) offers a pathway to 100+ institutions with asynchronous participation and network variability.

Computational requirements: All revision experiments were conducted on an NVIDIA RTX 3090 GPU, with each complete experimental configuration (dataset *×* attack type *×* distribution *×* $n{=}5$ seeds) requiring several hours of computation. This computational intensity is inherent to statistically rigorous federated learning evaluation with multi-seed replication, but also reflects the framework’s suitability for institutional deployment where GPU infrastructure is available. The overhead mitigation strategies discussed below reduce per-round costs substantially for production deployment.

Attack sophistication and adaptive attackers: While we evaluated five static Byzantine strategies and one dynamic schedule, real-world adversaries may employ adaptive strategies: alternating attacks, mimicking benign patterns, coordinated collusion, or zero-day exploits. To stress-test the most worrisome adaptive variant—a white-box attacker that explicitly targets the VAE fingerprint—we implemented a 20-step PGD attacker against a frozen snapshot of the server VAE (Table 20). The result has the principled, expected shape for any layered defense under a white-box attacker: *the detector degrades, but the aggregator does not*. Detection F1 dropped from *57.9%* (static control) to *33.1%* (adaptive), while final test accuracy was unchanged (64.35 vs *64.35%*). This is consistent with the broader literature on adaptive attacks against ML defenses: a sufficiently informed adaptive attacker can evade any static detector, but the trust-weighted aggregator still down-weights the malicious gradient enough to preserve the model. The natural hardening direction we identify is an *adversarially-trained VAE*—explicitly training the reconstruction head against PGD-generated adversarial gradients, analogous to adversarial training in image classification—which we plan as the next iteration of the framework. We also acknowledge that the adaptive attacker analysis used a single VAE-targeted variant; broader adaptive evaluations (Shapley-aware, attention-aware, multi-signal joint attackers) are an explicit future-work item. Continuous monitoring and adversarial red-teaming remain necessary for sustained protection in any deployment.

Trusted server assumption: OptiGradTrust assumes an honest-but-curious server, consistent with the threat model in FLTrust^24^, FLAME^25^, and recent trust evaluation frameworks such as TFL-DT^23^. This assumption is reasonable in regulated medical settings where the aggregation server is typically operated by a trusted coordinating institution (e.g., a university hospital or a national health authority) subject to audit and compliance requirements. However, server compromise remains a non-trivial concern in broader deployments: a malicious server could perform gradient inference attacks to reconstruct patient data, selectively exclude honest clients to bias the global model, or manipulate trust scores to favor specific participants. We identify three concrete mitigation pathways that are compatible with OptiGradTrust’s architecture: (i) *secure multi-party computation* (MPC) to distribute the trust scoring computation across multiple parties, preventing any single entity from observing raw gradient fingerprints^9^; (ii) *verifiable trust scoring* using zero-knowledge proofs, where the server publishes cryptographic proofs that trust weights were computed correctly from the declared fingerprint values, enabling client-side verification without revealing the scoring parameters; and (iii) *consensus-based model validation*, where a random subset of clients independently verify the aggregated model against their local validation sets before accepting the update. Integrating these mechanisms into the OptiGradTrust pipeline is a priority for future work; we note that the modular trust-scoring architecture (fingerprinting *→* attention *→* RL *→* aggregation) facilitates such extensions without redesigning the core framework.

Computational overhead and lightweight deployment: Empirical profiling (Table 15) reveals that total trust-scoring overhead is 10.4% of local training time (${\sim }$12.3 seconds per round on Alzheimer’s MRI with NVIDIA RTX 3090). Shapley value computation accounts for 9.3% of per-round time—the dominant cost—while all other eight trust components combined add less than 0.2%. This overhead is substantially lower than our initial theoretical estimate and is acceptable for institutional deployment. Three mitigation strategies can further reduce costs:

(1) Adaptive Shapley activation. Since Shapley is the dominant cost component (9.3%), activating it only when anomaly indicators (cosine, L2, sign-consistency) exceed thresholds can reduce per-round overhead to under 1% during benign periods. The ablation analysis shows that without Shapley, accuracy drops by ${\sim }$10pp; therefore, adaptive activation provides a principled cost–accuracy trade-off where Shapley engages only when the trust module detects anomalous gradient patterns. VAE fingerprinting adds only 40ms per round (0.03%) and can similarly be activated on demand with negligible baseline cost.

(2) Gradient quantization and communication compression. Applying QSGD^50^ with 8-bit quantization reduces gradient communication volume by *4×*. Since trust scoring operates on gradient *structure* (directions, norms, signs) rather than exact magnitudes, quantization has minimal impact on fingerprint quality. Combining quantization with top-*k* sparsification (transmitting only the largest 10% of gradient coordinates) can further reduce communication to near-baseline levels.

(3) Hierarchical aggregation for large federations. For consortia with 50+ institutions, a two-level hierarchy can be employed: regional aggregation nodes perform local trust-weighted aggregation, then forward pre-filtered updates to the central server. This reduces the central server’s per-round complexity from $O(N \cdot C_{\text {trust}})$ to $O(R \cdot C_{\text {trust}})$ where *R ≪ N* is the number of regions, achieving near-linear scalability.

RL stability and trust convergence: We provide a convergence analysis for the RL-driven trust weighting under Byzantine adversarial conditions. Let $Q^*(s,a)$ denote the optimal Q-function for the trust assignment MDP, and let $Q_t(s,a)$ be the estimate at round *t*. Under our DDQN formulation with soft target updates (rate *ρ =0.01*), replay buffer of size *B=1000*, and *ε*-greedy exploration ($\epsilon _t = \max (0.1, 1.0 \times 0.995^t)$), the Bellman error satisfies:

1 $$\begin{aligned} \mathbb {E}\Vert Q_t - Q^*\Vert _\infty \le (1 - \rho )^t \Vert Q_0 - Q^*\Vert _\infty + \frac{\rho \cdot \sigma _R^2}{1 - \gamma _R(1-\rho )}, \end{aligned}$$

where $\sigma _R^2$ is the reward variance and $\gamma _R=0.99$ is the discount factor. The first term decays geometrically, ensuring asymptotic convergence. The second term bounds the steady-state error, which remains small when reward variance is controlled—precisely the role of our reward shaping coefficients $(\alpha ,\beta ,\gamma ,\delta )=(1.0, 2.0, 3.0, 0.5)$.

Three design choices ensure practical stability: (i) reward shaping that prioritizes false negative minimization (coefficient *γ =3.0* in Eq. 22), critical for medical safety where failing to detect a malicious client is more dangerous than a false alarm; (ii) soft blending (*τ =0.3* in Eq. 21) that prevents abrupt trust changes by constraining the RL action influence, maintaining continuity between rounds and ensuring that the attention-based prior anchors the final trust weights; and (iii) DDQN target network updates every 10 rounds that decouple the target from rapidly changing estimates, reducing overestimation bias inherent in standard DQN^43^.

RL benefit-to-complexity ratio: Under static attack ablation, removing RL has negligible accuracy impact (*+0.08*pp on Alzheimer’s MRI), which is expected because static attacks do not exercise RL’s core capability—*temporal adaptivity*. The dynamic phase-transition experiment (Table 14) provides the dedicated evaluation: under a surprise switch from benign to scaling*×*20 at round 13, the RL component reduces detection F1 standard deviation by 12% (10.69% *→* 9.39%) and OptiGradTrust provides *2.3×* more stable accuracy than FedAvg. The RL’s contribution is *detection consistency and operational stability* rather than peak accuracy improvement—a meaningful benefit for safety-sensitive research deployments where reliable, round-to-round malicious-client identification is essential for accountability and auditability (clinical use specifically requires the additional prerequisites listed below). RL complements the dual-attention module on different axes: attention determines *which fingerprint signals* to emphasize for each gradient snapshot, while RL determines *how aggressively* to adjust trust across the temporal dimension^44^. The overhead is negligible (${\sim }$10ms per round, 0.01% of total), and the soft blending parameter ($\tau {=}0.3$ in Eq. 21) ensures that the attention-based prior anchors the final trust weights, making RL a low-cost module that cannot destabilize the system. More complex dynamic scenarios (multiple transitions, longer runs, alternating attack types) may amplify RL’s benefit; characterizing this more fully is a direction for future work.

DDQN discrete action space: The DDQN agent operates over a discrete action space $\mathcal {A} = \{0.0, 0.2, 0.4, 0.6, 0.8, 1.0\}^{|S_t|}$ (Eq. 21). While a continuous action space could provide finer-grained trust weight adjustments, the discrete formulation was chosen for training stability and sample efficiency in the low-data FL regime (10–50 clients, 25–30 rounds). The 0.2-step granularity, combined with soft blending ($\tau {=}0.3$), yields effective trust resolution of 0.06 per step—sufficient to differentiate honest from malicious clients. Extending to continuous action spaces via policy gradient methods (e.g., SAC, TD3) is a viable future direction for larger federations where finer discrimination may be beneficial.

VAE interpretability and distributional anomaly detection: The attack-specific ablation now provides direct evidence for the VAE’s role. Under scaling attack, VAE removal has zero accuracy impact (Table 10)—expected because scaling preserves gradient structure and L2 norm already captures the anomaly. Under Gaussian noise injection ($\sigma {=}15.0$), however, VAE removal causes a substantial accuracy drop of *-3.60*pp (Table 11), confirming that the VAE reconstruction error^41,42^ is the primary signal enabling defense against noise-based attacks. This attack-specific validation directly demonstrates the defense-in-depth design: each component contributes under its targeted threat type. Beyond detection, VAE provides *interpretability*: the reconstruction error offers a visual and quantitative indicator of gradient anomaly that clinicians and auditors can inspect, supporting regulatory transparency requirements. In practical deployment, we recommend adaptive activation (enable VAE when anomaly indicators exceed predefined thresholds), which provides both on-demand interpretability and noise-attack coverage at negligible computational cost (40ms per round, 0.03% of total).

Detection gap for subtle attacks, and the honest non-IID sign-flipping cell: While OptiGradTrust achieves near-perfect detection for scaling attacks (F1*\ge*0.98) and strong noise-injection detection (F1*\ge*0.78), formal detection of sign-flipping and label-flipping attacks remains limited (F1<0.50, Table 5). The sign-flipping ablation (Table 12) confirms this: formal detection F1 is 0% across all configurations, which is architecturally expected because sign-flipping preserves gradient magnitude—a known limitation of magnitude-based Byzantine detection consistent with prior analyses^11,13^. The R3 multi-seed table (Table 18) makes one further honest finding explicit: in the single Non-IID + sign-flipping cell, FLTrust ($40.7 \pm 25.8\%$ F1) outperforms OptiGradTrust ($13.3 \pm 18.9\%$ F1) on detection, with both methods having statistically indistinguishable accuracy (*64.4%* vs *64.2%*). This is the only one of 18 paired comparisons in the multi-seed panel where a baseline visibly beats OptiGradTrust on F1, and we attribute it to the specific alignment of FLTrust’s ReLU-cosine head with magnitude-preserving sign-flips by a small malicious population: the sign-flipped gradient has a strongly negative cosine with the reference, which is precisely what FLTrust’s clipping function thresholds. Crucially, model accuracy is *fully preserved* despite this detection gap: all sign-flipping configurations achieve 64–*66%*, demonstrating zero accuracy degradation compared to benign federated learning. This robustness-without-detection arises from implicit suppression via trust-weighted aggregation and the benign client majority, and is a key benefit of the optimizer-security co-design. We emphasize that detection claims are attack-specific: OptiGradTrust provides strong formal detection for high-signal attacks (scaling, noise) and implicit aggregation-level robustness for magnitude-preserving attacks (sign-flipping, label-flipping). Improving formal detection for subtle attacks—potentially via distributional fingerprinting, temporal trajectory analysis, or hybridizing the FLTrust-style ReLU-cosine signal as a seventh fingerprint dimension—is an important future direction.

Parameter sensitivity: Reward coefficients $(\alpha ,\beta ,\gamma ,\delta )$ and soft fusion *τ* are critical; asymmetric weighting reflects the principle that false negatives (undetected malicious updates) pose greater risk than false alarms. $\tau \in [0.2, 0.4]$ balances RL adaptivity with stability; sensitivity over $\tau \in \{0.1,\ldots ,0.5\}$ gave <1.2pp accuracy variation on Alzheimer’s MRI. Monotonic convergence and reduced variance versus FedAvg (Results) validate the design.

Privacy considerations and compatibility with secure aggregation/DP: A reviewer correctly observed that sharing per-client gradient *statistics*—norms, sign-consistency ratios, peer cosine similarities, and Monte Carlo Shapley estimates—can in principle leak information about a client’s local data distribution, even though the raw gradient itself never leaves the institution. We address this in three explicit layers.

- Compatibility with differential privacy (DP): OptiGradTrust does not impose any structure that prevents clients from clipping and noising their gradients with calibrated DP noise (e.g., DP-SGD) before submission, so the framework *may be compatible* with client-side DP. Because the trust signals operate on gradient *structure* (directional cosine, sign agreement, magnitude) rather than coordinate-wise exact values, moderate DP noise may preserve some fingerprint structure, but the exact tolerance depends on the noise scale, dataset, and attack type and remains to be quantified empirically; this experimental analysis has not been performed in the present manuscript. Aggressive noise (*ε < 1*) is expected to degrade fingerprint quality, particularly the VAE reconstruction-error and peer-cosine signals; quantifying the *ε*–detection-F1 trade-off empirically is a clear next step. The Shapley estimator is the most sensitive component because it relies on validation-loss queries; for strict DP guarantees, a noisy-Shapley variant^51^ *could in principle be adopted*, with the accuracy and privacy trade-offs to be characterized in future work.
- Compatibility with secure aggregation: The dominant fingerprint signals—$L_2$ norm, cosine similarity to a server reference, sign-consistency, and peer-pair cosine similarities—can be evaluated inside a secure-aggregation pipeline (additive secret-sharing, threshold homomorphic encryption^9,19^, or pairwise masking) by computing the inner products and norms in the encrypted domain; the server only sees the aggregated trust-weighted update. Shapley sub-coalition evaluations and the VAE reconstruction error currently require gradient-domain access and would need a secure-multi-party-computation (MPC) variant for full secure-aggregation compatibility; this is a concrete research direction with established cryptographic primitives.
- Gradient-inference and membership-inference risk surface: Honest-but-curious server-side gradient-inference attacks (e.g., gradient inversion, membership inference) operate on the gradient *itself*, not on the fingerprint scalars; from this perspective the fingerprint adds at most a few hundred bits of side-channel information per round, which is small relative to the millions of gradient coordinates already sent. Nonetheless, the trust-score history and Shapley trajectory *do* carry distributional information; we recommend that production deployments combine OptiGradTrust with (i) gradient clipping at the client, (ii) DP noise calibrated to the consortium’s privacy policy, and (iii) verifiable trust scoring via cryptographic commitments so that the server publishes a proof that the aggregated weights match the declared fingerprints, without revealing the per-client scoring parameters.

In summary, OptiGradTrust is *conceptually compatible* with differential privacy and *partially compatible* with secure aggregation in its present form—neither has been experimentally demonstrated in this manuscript—and the architectural changes required for full MPC-style secure aggregation are well-scoped and identified as priority follow-up work. The framework’s explainability features (VAE reconstruction visualization, Shapley attribution) support transparency requirements consistent with FDA AI/ML guidance and the EU Medical Device Regulation, but we make no claim that the present system is regulator-ready. Decentralized trust architectures^52^ may offer complementary approaches for establishing inter-institutional trust in future deployments.

Clinical research implications and what would be required for clinical use: OptiGradTrust’s modular architecture is intended to support heterogeneous *research* consortia where institutions differ in resources and privacy policies. Small clinics could, in principle, train using standard local loops, while large academic centers handle trust aggregation on multi-GPU servers. This asymmetric design aims to enable broad participation in collaborative *research*. We are explicit about the gap between the present evidence and any clinical claim: with only *∼*87 OASIS test samples and three seeds on the Alzheimer’s MRI benchmark, the present evidence is sufficient to support a methods-level contribution and benchmarking study, but *not* sufficient to support claims about diagnostic accuracy, clinical utility, or patient-level decision support. Concretely, we view the following as prerequisites for any clinical-deployment claim: (i) validation on at least one large multi-center brain-MRI cohort with documented site-level metadata (e.g., ADNI, UK Biobank subsets, BraTS), (ii) per-site, per-scanner, per-vendor, and per-subgroup (age, sex, field strength) performance reporting with calibration curves, (iii) prospective evaluation under a documented quality management system, (iv) regulatory review under the relevant medical-device frameworks (FDA SaMD, EU MDR), and (v) bias and fairness audits on the target patient population. Multi-center cohort validation requires institutional data-use agreements that were beyond the scope of this revision but is a planned, prioritized follow-up study.

Baseline selection and comparison scope: The overall baseline panel reported in this manuscript comprises eight methods: six Byzantine-robust aggregators—Krum, FLTrust^24^, FLAME^25^, FLGuard^26^, RFA^13^, and SignGuard^14^—together with the standard FedAvg and the heterogeneity-aware FedBN as non-robust accuracy reference points. These methods span the major paradigms: pure aggregation (FedAvg, FedBN; reference baselines), distance-based selection (Krum), trust-scoring via reference gradients (FLTrust), geometric-median robust point estimation (RFA), sign-clustering filtering (SignGuard), clustering-based filtering (FLAME), multi-signal aggregation (FLGuard), and our six-signal trust framework (OptiGradTrust). FLAME and FLGuard appear in the original-configuration comparison (Table 3); RFA and SignGuard were added in the multi-seed extension (Section 2.14). This provides representative coverage of the major Byzantine-robust FL paradigms. More recent methods such as DnC (Divide-and-Conquer)^53^ and Centered Clipping^54^ were considered but excluded for specific reasons: DnC operates on spectral decomposition of gradient updates, which provides complementary but not directly comparable defense properties to our trust-based approach; Centered Clipping addresses coordinate-wise gradient manipulation, overlapping primarily with our L2 norm and sign-consistency signals. Both methods focus on aggregation robustness without per-client detection or trust assessment, making direct comparison less informative for evaluating OptiGradTrust’s unique detection and accountability capabilities. We acknowledge that including these and additional 2024–2025 medical-FL baselines (e.g., recent personalized-FL defenses for medical imaging) would further strengthen the comparison and consider this an important direction for future work.

Optimizer comparison: FedADMM: In the optimizer comparison (Fig. 5), FedADMM^49^ shows notably lower performance than other optimizers. This is attributable to FedADMM’s reliance on dual variable updates that assume relatively homogeneous client objectives; under our Byzantine attack scenario with 30% malicious clients and non-IID data, the dual variables accumulate adversarial bias, leading to oscillatory convergence and suboptimal final accuracy. FedADMM was designed for consensus optimization in cooperative settings and lacks the adversarial robustness mechanisms present in FedBN-P’s masked proximal design.

Future directions: Next steps include validation on large federated medical cohorts (FeTS, ADNI, UK Biobank), extension to 100+ clients with asynchronous participation, improved detection of subtle attacks via distributional fingerprinting, and cross-modal trust fusion (MRI, EEG, omics, EHR). The rapid progress in foundation models for medical image segmentation^55^ and large language models in biomedicine^56^ opens opportunities for trust-aware federated fine-tuning of foundation models, where Byzantine robustness becomes increasingly critical as model capacity grows. Efficient GPU cluster serving^57^ may enable scalable deployment of computationally intensive trust-scoring pipelines. Federated synthetic data, on-device inference, and multi-agent RL for threat intelligence are also promising directions.

In summary, multi-signal gradient fingerprinting with RL-driven trust weighting offers a viable path to Byzantine-robust FL under heterogeneous medical-research data. Attack-specific ablation experiments validate the defense-in-depth design, with each component contributing under its targeted threat type: VAE for noise injection, Shapley for functional accuracy under scaling, RL for detection consistency under dynamic threats, and aggregation-level robustness for magnitude-preserving attacks. The third-round additional analyses (Section 2.14) further provide: a formal failure-mode characterization across attack budgets, computational and communication overhead measured against four representative baselines (with *+8.8%* wall-clock and identical communication cost relative to FedAvg), three-seed paired-test validation on 24 headline cells with effect sizes alongside *p*-values, a white-box adaptive-attacker stress-test that confirms the layered defense preserves accuracy even when the detector is partially evaded, an explicit RL-vs-rule-based ablation suggesting that the learned trust-head components, particularly the dual-attention pathway, contribute substantially to more stable detection under the evaluated dynamic schedule, and two additional Byzantine-robust baselines (RFA, SignGuard). Limits include small clinical cohort size (*∼*87 OASIS test samples), simulated federation, single adaptive-attacker variant, $n{=}3$ statistical power for paired tests, peak GPU memory of *4.5×* FedAvg, and limited formal detection of subtle attacks (with one Non-IID sign-flipping cell where FLTrust outperforms our method on F1, accuracy unchanged); validation on larger federated cohorts, broader adaptive evaluations, adversarially-trained VAE fingerprints, integration with secure-aggregation primitives, and per-site/subgroup analyses on multi-center data remain explicit future-work items. These results support OptiGradTrust as a methodological foundation for secure, privacy-aware multi-institutional medical-AI *research*, while we make no claim of clinical-deployment readiness.

## Methods

### Problem formulation and threat model

We consider federated learning with *N* clients (e.g., hospitals), each holding private datasets $\mathcal {D}_k$ that cannot be shared due to privacy regulations. The goal is to minimize population risk without centralizing data:

2 $$\begin{aligned} \theta ^* = \arg \min _{\theta } F(\theta ) = \sum _{k=1}^{N} \frac{|\mathcal {D}_k|}{|\mathcal {D}|}\, F_k(\theta ), \end{aligned}$$

where $F_k(\theta )$ is the empirical loss at client *k* and $|\mathcal {D}|{=}\sum _k |\mathcal {D}_k|$.

Following established Byzantine-robust federated learning studies^13,21,24–26^, we evaluate under two threat configurations: (i) the original configuration with *30%* malicious clients performing scaling (*× 10*), partial scaling (50% dimensions, *× 5*), sign flipping (*λ =-1*), Gaussian noise ($\sigma \in \{0.5, 1.0\}$), or label flipping ($p_{\text {flip}}=0.5$); and (ii) a stronger revision configuration with *40%* malicious clients, scaling (*× 30*), Gaussian noise ($\sigma {=}15.0$), and label flipping ($p_{\text {flip}}=0.9$), designed to stress-test robustness under more aggressive adversarial conditions. The stronger configuration follows recent findings on enhanced model poisoning^21^ demonstrating that higher malicious ratios and attack intensities better differentiate robust defenses from vulnerable baselines. To address these attacks, OptiGradTrust combines robust optimization, gradient-anomaly analysis, adaptive trust learning, and privacy-preserving aggregation.

Formal threat model: We adopt the following formal specification, summarized in Table 22, which we use consistently in all evaluations and in the adaptive-attacker analysis (Section 2.14).

**Table 22:** Formal threat model for OptiGradTrust evaluations. Each axis is specified explicitly to enable principled comparison with prior Byzantine-robust FL work and with future evaluations.

Axis	Assumption used in this work
Adversary’s knowledge	White-box knowledge of the federated training algorithm, the model architecture, and the high-level fingerprinting pipeline (six fingerprint dimensions). The adversary does *not* have access to the server-side validation data $D_{\textrm{val}}$, the trained VAE weights and replay buffer, the dual-attention parameters $(W_g, W_f, b_w, u)$, or the DDQN policy parameters; nor to any honest client’s local data or ephemeral random seeds. The adaptive attacker in Section 2.14 (R3-D) is granted additional white-box access to a frozen snapshot of the server VAE, representing a leak of the most sensitive component
Adversary’s capability	Each malicious client fully controls its own local training (data, label, optimization, gradient post-processing) and may submit any update $g'_k \in \mathbb {R}^d$ in place of the honest gradient. Adversaries may craft updates after observing the global model $\theta ^t$ (which all clients receive) but cannot observe other clients’ submitted gradients before submitting their own (no late-mover advantage)
Collusion	Malicious clients may collude with one another off-band (shared strategy, shared $\theta ^t$, shared random seed). Honest clients do not collude. The server does not collude with the adversary
Fraction over time	The malicious fraction *f ∈ [0,1]* is constant across rounds in static-attack experiments and varied dynamically in the schedule analysis (benign for rounds 1–12, attack for rounds 13–25 in Table 14). We evaluate $f \in \{10\%, 20\%, 30\%, 40\%, 60\%\}$ across experiments, with *30%* as the default operating point
Adaptivity	Two adaptivity levels are evaluated: (i) *static* adversary that fixes a single attack family and intensity for the entire run (Sections 2.2–2.6); (ii) *round-adaptive* adversary that switches attack family or intensity at a known round (Table 14). For the white-box adaptive attacker (Section 2.14, R3-D), each malicious client additionally runs an inner-loop projected-gradient optimization against a frozen snapshot of the server VAE before submitting each update
Server model	Honest-but-curious. The server faithfully executes the published aggregation protocol but may attempt to infer client-side information from the gradient fingerprints it observes; we discuss compatibility with secure-aggregation, multi-party computation, and differential-privacy hardening in the Discussion
Communication channel	Authenticated, integrity-protected channels between each client and the server (standard TLS); message tampering by network adversaries is out of scope

This formal model aligns with FLTrust^24^, FLAME^25^, and recent trust-evaluation frameworks^22,23^ on the honest-but-curious server assumption, while being more explicit on adversary knowledge, collusion, fraction-over-time, and adaptivity than typical Byzantine-robust FL papers.

### FedBN-P optimizer: masked proximal hybrid

To stabilize training under heterogeneous medical data distributions and Byzantine attacks, OptiGradTrust employs FedBN-P, which combines localized batch normalization^32^ with proximal regularization^33^. Each client *k* minimizes a regularized local objective at round *t*:

3 $$\begin{aligned} \mathcal {L}_k(\theta ) = F_k(\theta ) + \frac{\mu }{2}\Vert \theta - \theta ^t\Vert ^2, \end{aligned}$$

where $F_k(\theta )$ is the empirical loss, $\theta ^t$ are global parameters from the previous round, and *μ >0* is the proximal coefficient.

Relation to prior work and co-design rationale: Hwang et al.^38^ explored similar hybrid optimizers for medical federated learning under statistical heterogeneity. Our FedBN-P differs in both intent and integration: it is *co-designed* with OptiGradTrust’s trust-aware aggregation (six-signal fingerprinting, attention, RL control), enabling the optimizer to act as a stability lever specifically under Byzantine behaviors rather than honest-only regimes. The BatchNorm locality component preserves domain-specific statistics (critical when hospitals use different MRI scanners), while the masked proximal term stabilizes non-BN parameters against adversarial drift—a synergy whose quantitative benefits under adversarial conditions are analyzed in the Discussion. This optimizer-security co-design distinguishes FedBN-P from optimization-focused hybrids such as Hwang et al.^38^ and motivates its inclusion as an integral part of the OptiGradTrust defense pipeline rather than a standalone optimizer.

Masked-proximal definition: We partition model parameters into two disjoint sets: $\mathcal {B}$ (BatchNorm parameters) and $\bar{\mathcal {B}}$ (all non-BN parameters). The masked proximal objective is:

4 $$\begin{aligned} L_k(\theta ) \;=\; F_k(\theta )\;+\;\frac{\mu }{2}\,\big \Vert \theta _{\bar{\mathcal {B}}} - \theta ^{t}_{\bar{\mathcal {B}}} \big \Vert _2^2, \end{aligned}$$

where the proximal term excludes BN parameters by default, consistent with FedBN’s locality assumption, reducing client drift in non-IID regimes while preserving domain-specific normalization statistics.

After *E* epochs of stochastic gradient descent with learning rate *η*, the local update is:

5 $$\begin{aligned} \theta _k^{t+1} \leftarrow \theta ^t - \eta \sum _{e=1}^{E} \nabla \mathcal {L}_k(\theta ), \end{aligned}$$

and the transmitted gradient update is:

6 $$\begin{aligned} g_k^t = \theta _k^{t+1} - \theta ^t. \end{aligned}$$

### Six-dimensional gradient fingerprinting

The trust assessment begins with a multi-signal fingerprint characterizing each client’s gradient update from complementary perspectives. Let $S_t$ denote participating clients at round *t*, $g_k^t \in \mathbb {R}^d$ the update from client *k*, and $g_{\text {ref}}^t$ a trusted reference gradient computed on a small server-side validation subset. Table 23 summarizes the six fingerprint dimensions, each formally defined in the equations below.

**Table 23:** Six-dimensional gradient fingerprint: notation and roles. Each signal targets a distinct anomaly axis; together they form the input vector $f_k^t \in \mathbb {R}^6$ to the dual-attention + RL trust head.

#	Symbol	Definition (Eq. ref)	Primary anomaly axis
1	$f_1(g_k^t)$	VAE reconstruction error (Eq. 7)	Distributional/off-manifold updates (e.g., Gaussian noise injection, novel attack types)
2	$f_2(g_k^t)$	Cosine similarity to server reference (Eq. 8)	Directional shifts relative to a trusted gradient (e.g., sign-flipping, partial reversal)
3	$f_3(g_k^t)$	Mean cosine similarity to peers (Eq. 9)	Coordinated collusion or majority deviation
4	$f_4(g_k^t)$	Gradient $L_2$ norm (Eq. 10)	Magnitude anomalies (e.g., scaling attacks)
5	$f_5(g_k^t)$	Coordinate-wise sign-consistency ratio (Eq. 11)	Partial sign manipulation, sign-flipping (in benign-minority regimes)
6	$f_6(g_k^t)$	Smoothed Monte Carlo Shapley contribution (Eqs. 12–14)	Functional contribution: any attack that degrades validation loss regardless of gradient structure

1. VAE reconstruction error: A variational autoencoder trained on historical benign gradients provides a distributional model. The reconstruction error is:
  7 $$\begin{aligned} f_1(g_k^t) \;=\; \big \Vert \, g_k^t - \textrm{VAE}(g_k^t) \,\big \Vert _2^2, \end{aligned}$$
  where large values indicate off-manifold behavior.
2. Cosine similarity to reference: Directional consistency with the trusted reference:
  8 $$\begin{aligned} f_2(g_k^t) \;=\; \frac{g_k^t \cdot g_{\text {ref}}^t}{\Vert g_k^t\Vert _2 \,\Vert g_{\text {ref}}^t\Vert _2}. \end{aligned}$$
3. Peer consensus similarity: Agreement with same-round peers:
  9 $$\begin{aligned} f_3(g_k^t) \;=\; \frac{1}{|S_t|-1}\!\!\sum _{\begin{array}{c} j \in S_t \\ j \ne k \end{array}} \frac{g_k^t \cdot g_j^t}{\Vert g_k^t\Vert _2 \,\Vert g_j^t\Vert _2}. \end{aligned}$$
4. L2 norm: Magnitude highlights scaling attacks:
  10 $$\begin{aligned} f_4(g_k^t) \;=\; \Vert g_k^t\Vert _2. \end{aligned}$$
5. Sign-consistency ratio: Coordinate-wise sign agreement:
  11 $$\begin{aligned} f_5(g_k^t) \;=\; \frac{1}{d}\sum _{i=1}^{d} \mathbb {I}\!\Big [\textrm{sign}\!\big (g_k^t[i]\big ) = \textrm{sign}\!\big (g_{\text {ref}}^t[i]\big )\Big ]. \end{aligned}$$
6. Shapley-based contribution: Functional contribution quantified by Monte Carlo Shapley approximation^35,36^. For permutation *π* of $S_t$, define $S_{\pi }^{-k}$ as clients preceding *k* and $S_{\pi }^{+k} = S_{\pi }^{-k} \cup \{k\}$. The marginal contribution is:
  12 $$\begin{aligned} \Delta _\pi (k) \;=\; L\!\big (\theta _{S_{\pi }^{-k}}, D_{\textrm{val}}\big ) \;-\; L\!\big (\theta _{S_{\pi }^{+k}}, D_{\textrm{val}}\big ), \end{aligned}$$
  averaged over *M* random permutations:
  13 $$\begin{aligned} \phi _k^t \;=\; \frac{1}{M}\sum _{m=1}^{M} \Delta _{\pi _m}(k), \end{aligned}$$
  with exponential smoothing for temporal stability:
  14 $$\begin{aligned} f_6(g_k^t) \;=\; \beta \, \phi _k^t \;+\; (1-\beta )\, f_6(g_k^{t-1}), \quad \beta \in (0,1). \end{aligned}$$
  The six-dimensional fingerprint is:
  15 $$\begin{aligned} f_k^t = \big [\, f_1(g_k^t),\; f_2(g_k^t),\; f_3(g_k^t),\; f_4(g_k^t),\; f_5(g_k^t),\; f_6(g_k^t) \,\big ]. \end{aligned}$$

### Dual attention and reinforcement learning

The fingerprint features are processed jointly with raw gradient structure by a dual-attention module that emphasizes the most informative evidence at each round and client. The gradient vector is partitioned into *P* contiguous chunks; per chunk *p*, we compute local self-attention:

16 $$\begin{aligned} \text {LocalAttn}_p(Q_p, K_p, V_p) = \text {softmax}\!\left( \frac{Q_p K_p^\top }{\sqrt{d_a}}\right) V_p, \end{aligned}$$

followed by global attention over chunks:

17 $$\begin{aligned} \textrm{GlobalAttn}(Q,K,V) \;=\; \textrm{softmax}\!\Big (\tfrac{QK^\top }{\sqrt{d_a}}\Big )\,V, \end{aligned}$$

yielding gradient-side representation $\tilde{g}_k^t$.

In parallel, an attention layer operates on $f_k^t$ to infer feature importances:

18 $$\begin{aligned} \alpha _k^t \;=\; \textrm{softmax}(W_f f_k^t + b_f), \quad f_{k,\textrm{attn}}^t \;=\; \alpha _k^t \odot f_k^t. \end{aligned}$$

Fusion produces the joint representation:

19 $$\begin{aligned} h_k^t \;=\; \sigma \!\big ( W_g \tilde{g}_k^t + W_f^{\textrm{fuse}} f_{k,\textrm{attn}}^t + b_{\textrm{fuse}}\big ), \end{aligned}$$

from which a prior trust weight is computed:

20 $$\begin{aligned} \hat{w}_k^t \;=\; \textrm{sigmoid}(u^\top h_k^t + b_w) \in (0,1). \end{aligned}$$

Formal MDP specification for the RL trust controller: The DDQN agent^43^ that refines the attention-based trust prior is trained as a finite-horizon Markov Decision Process $\mathcal {M} = (\mathcal {S}, \mathcal {A}, P, R, \gamma _R, H)$ defined as follows:

- State space $\mathcal {S}$: at round *t*, the state is $s_t = \big ( \{h_k^t\}_{k\in S_t},\; \textrm{history}_t \big )$, where $h_k^t \in \mathbb {R}^{64}$ is the per-client fused representation from the dual-attention module (Eq. 19) and $\textrm{history}_t$ is a fixed-length window of the four most recent rounds’ trust weights and reward signals. The state dimension is therefore $64 \cdot |S_t| + 4 \cdot (|S_t| + 1)$.
- Action space $\mathcal {A}$: discrete per-client trust adjustment, $\mathcal {A} = \{0.0,\,0.2,\,0.4,\,0.6,\,0.8,\,1.0\}^{|S_t|}$. The discrete formulation is chosen for sample efficiency in the low-data FL regime (25–30 rounds, 10–50 clients); extensions to continuous policies (SAC, TD3) are a future direction.
- Transition kernel *P*: deterministic given the gradient updates and aggregator: $s_{t+1}$ is computed by running the next round of FL with trust weights $w_t$ derived from $a_t$ via Eq. 21. Stochasticity arises from client mini-batch sampling, attacker behavior, and Shapley permutations.
- Reward function *R*: one-step reward at round *t* (Eq. 22, defined below) combines accuracy improvement on the server-side validation set, false-positive and false-negative detection rates, and trust-weight variance, with coefficients $(\alpha ,\beta ,\gamma ,\delta ) = (1.0, 2.0, 3.0, 0.5)$ that asymmetrically penalize false negatives because failing to detect a malicious client is more dangerous than a false alarm in safety-sensitive medical settings.
- Discount factor $\gamma _R = 0.99$, encouraging the agent to optimize for long-horizon trust assignments rather than greedy per-round detection.
- Horizon *H = 25*–30 for benchmark experiments and *H = 25* for all R3 experiments.

The agent is trained with double Q-learning, soft target updates ($\rho {=}0.01$ per round), *ε*-greedy exploration ($\epsilon _t = \max (0.1, 1.0 \times 0.995^t)$), a replay buffer of 1000 experiences, batch size 32, and the target network refreshed every 10 rounds. Training-stability evidence across the three R3 seeds is reported in Table 14 (post-attack detection F1 standard deviation: *9.4%* for full RL versus *10.7%* for the no-RL baseline). The MDP formulation, together with the soft-blending parameter *τ* (Eq. 21), ensures that the RL action is bounded relative to the attention-based prior, so a misbehaving Q-network cannot produce arbitrarily large trust swings.

A Double Deep Q-Network (DDQN)^43^ agent refines the prior using state $s_t = \{h_k^t\}_{k\in S_t} \cup \textrm{history}_t$ and discrete action space $\mathcal {A} = \{0.0,\,0.2,\,0.4,\,0.6,\,0.8,\,1.0\}^{|S_t|}$. The final trust weights are:

21 $$\begin{aligned} w_k^t \,=\, \tau \, a_{t,k} \;+\; (1-\tau )\, \hat{w}_k^t, \end{aligned}$$

where $\tau \in [0,1]$ controls action strength.

The reward function prioritizes long-term robustness:

22 $$\begin{aligned} R_t \,=\, \alpha \cdot \Delta \!\textrm{ACC}_t \;-\; \beta \cdot \textrm{FPR}_t \;-\; \gamma \cdot \textrm{FNR}_t \;-\; \delta \cdot \textrm{Var}(w_t), \end{aligned}$$

with coefficients $(\alpha ,\beta ,\gamma ,\delta )=(1.0, 2.0, 3.0, 0.5)$ emphasizing false negative minimization.

Trust-weighted aggregation updates the global model:

23 $$\begin{aligned} \theta ^{t+1} \;=\; \theta ^t \;+\; \sum _{k\in S_t} w_k^t\, g_k^t. \end{aligned}$$

### Convergence theory

Under standard assumptions—(i) each local objective $F_k(\theta )$ is *L*-smooth and *μ*-strongly convex, (ii) bounded gradient variance $\mathbb {E}\Vert \nabla F_k(\theta )-\nabla F(\theta )\Vert ^2 \le \sigma ^2$, and (iii) trust weights $T(g_k^t)$ normalized so $\sum _k T(g_k^t)=1$—the expected gradient norm follows:

24 $$\begin{aligned} \mathbb {E}\Vert \nabla F(\theta ^{t+1})\Vert ^2 \;\le \; (1-\eta \mu )\,\mathbb {E}\Vert \nabla F(\theta ^{t})\Vert ^2 \;+\; \eta ^2 L^2 \sigma ^2 + \eta ^2 L^2 \Omega _{\text {Byz}}, \end{aligned}$$

where $\Omega _{\text {Byz}} = \mathbb {E}\big [\Vert \sum _k (T(g_k^t)-1/K)\,g_k^t \Vert ^2\big ]$ captures residual bias from malicious clients. Under 40% Byzantine ratio, the trust module ensures $\Omega _{\text {Byz}}\!\le \!0.25 \sigma ^2$ empirically, yielding stable descent.

### Implementation details

Network architectures: MNIST: 3-layer CNN (32-64-128 filters, 3*×*3 kernels, ReLU activation, 2*×*2 max pooling, dropout 0.25, fully connected 128*→*10). CIFAR-10 and Alzheimer’s MRI: ResNet-18^58^ with modifications for 4-class output (MRI) and standard 10-class output (CIFAR-10).

VAE architecture: Encoder: 3 fully-connected layers (input dim *d* *→* 512 *→* 256 *→* 128), latent dimension 64. Decoder: symmetric structure (128 *→* 256 *→* 512 *→* *d*). Trained via ELBO maximization on first 5 rounds of benign gradients, updated every 20 rounds in benchmark experiments. For 8-round clinical validation runs, the VAE is warm-started from benchmark pre-training and held fixed (see Results).

Attention architecture: Gradient partitioned into *P=16* chunks. Local attention: 4 heads, key dimension $d_a=64$. Global attention: 2 heads, $d_a=128$. Feature attention: single layer, output dimension 6 (matches fingerprint size).

DDQN architecture: State embedding: fully-connected 256*→*128*→*64. Q-network: 2-layer MLP (64*→*128*→* $|\mathcal {A}|$). Target network updated every 10 rounds with soft update rate 0.01 (benchmark experiments; warm-started and fixed for 8-round clinical validation). Replay buffer size 1000 experiences, batch size 32, discount factor $\gamma _R=0.99$, *ε*-greedy exploration (start=1.0, end=0.1, decay=0.995).

Hyperparameters: Learning rate $\eta =1\times 10^{-4}$, weight decay $5\times 10^{-5}$, batch sizes 64 (MNIST/CIFAR-10) and 16 (MRI), local epochs *E=5* (MNIST), *E=8* (CIFAR-10/MRI), proximal coefficient *μ =0.01*, Shapley smoothing *β =0.8*, adaptive $M \in \{50, 200\}$ based on outlier detection, action blend *τ =0.3*.

Computational environment: Experiments conducted on NVIDIA RTX 3090 GPU (24 GB VRAM), Intel Core i9-12900K CPU (16 cores), 64 GB RAM. Average wall-time per round: MNIST  45s, CIFAR-10  120 s, Alzheimer’s MRI  180 s (including trust computation). Parallelization via PyTorch DataParallel for client updates and multiprocessing for Shapley permutations.

## Supplementary Information

Supplementary Information.

## Data Availability

This study used only publicly available, de-identified datasets. The MNIST dataset was accessed via the TensorFlow/Keras canonical release (https://storage.googleapis.com/tensorflow/tf-keras-datasets/mnist.npz). CIFAR-10 is available at https://www.cs.toronto.edu/~kriz/cifar.html. The Alzheimer’s MRI dataset is a public Kaggle resource (https://www.kaggle.com/datasets/lukechugh/best-alzheimer-mri-dataset-99-accuracy). The OASIS (Open Access Series of Imaging Studies) cross-sectional MRI dataset is available at https://www.oasis-brains.org/. All datasets were used in accordance with their respective licenses. No new human data were collected; OASIS data are deÃ¢â‚¬â€˜identified and released under open-access terms.

## Code availability

The code implementing OptiGradTrust (training loops, six-dimensional fingerprinting, dual-attention, DDQN trust controller, FedBN-P optimizer, and all evaluation scripts) is publicly available at https://github.com/mohammadkarami79/OptiGradTrust. The implementation uses Python 3.9, PyTorch 1.12 (and tested up to PyTorch 2.7 on the R3 experiments), and standard scientific Python libraries (NumPy, SciPy, Matplotlib). **Reproducibility commitment.** The repository includes: (i) comprehensive documentation and configuration files for every experiment reported in the paper (original-configuration runs, strengthened-configuration runs, and the six R3 additional analyses described in Section 2.14); (ii) deterministic seed lists ($\{42, 123, 456\}$ for R3, plus $\{42, 123, 456, 789, 1024\}$ for the strengthened-configuration multi-seed runs) so that every reported number can be reproduced; (iii) the exact data splits used for OASIS, Alzheimer’s MRI, MNIST, and CIFAR-10; (iv) trained VAE checkpoints for the Alzheimer’s MRI benchmark; (v) per-experiment raw CSV / JSON artifacts for the R3 analyses; and (vi) an environment.yml pinning all package versions. Upon acceptance we will additionally release a Dockerfile encapsulating the full software stack.
