# Supplementary material for: Byzantine robust federated learning for heterogeneous brain MRI using multisignal gradient fingerprinting and adaptive trust aggregation
Source: Sci Rep. 2026 Jun 4;16:25604. doi: 10.1038/s41598-026-55855-5 (PMC13478148; doi:10.1038/s41598-026-55855-5)
Supplement: Supplementary file 1 [file 41598_2026_55855_MOESM1_ESM.pdf]

Supplementary Information for:

Trust-aware Byzantine-robust federated learning for non-IID brain MRI via multi-signal gradient fingerprinting and adaptive aggregation

Mohammad Karami, Hamed Kebriaei, Fatemeh Ghassemi, Hamid Azadegan

1 Supplementary Methods

1.1 Detailed Algorithm Pseudocode

We present the OptiGradTrust framework as three modular algorithms for clarity: (S1) FedBN-P client-side training, (S2) six-dimensional gradient fingerprinting, and (S3) trust-weighted aggregation with RL adaptation.

Algorithm S1: FedBN-P Client-Side Local Training

Algorithm 1 FedBN-P Client-Side Training (per round t)

Input: Global parameters θ^t, local dataset $\mathcal{D}_k$, proximal coefficient μ, learning rate η, local epochs E
Initialize: $\theta_k^{(0)} \leftarrow \theta^t$
for epoch $e = 1$ to E **do**
  Sample mini-batch $\mathcal{B}_k \sim \mathcal{D}_k$
  Compute loss: $\mathcal{L}_k = F_k(\theta_k^{(e)}) + \frac{\mu}{2}\|\theta_k^{(e)} - \theta^t\|^2$ *(proximal regularization)*
  Update: $\theta_k^{(e+1)} \leftarrow \theta_k^{(e)} - \eta\nabla\mathcal{L}_k$
end for
Compute gradient: $g_k^t = \theta_k^{(E)} - \theta^t$
Output: Send g_k^t to server (keep BatchNorm statistics local)

Algorithm S2: Six-Dimensional Gradient Fingerprinting

Algorithm 2 Gradient Fingerprinting (server-side, per round t)

Input: Client gradients $\{g_k^t\}_{k\in S_t}$, reference gradient g_{ref}^t, VAE model, Shapley sample size M
for each client $k \in S_t$ **do**
  $f_1(g_k^t) \leftarrow \|g_k^t - \text{VAE}(g_k^t)\|_2^2$ *(VAE reconstruction error)*
  $f_2(g_k^t) \leftarrow \text{cosine}(g_k^t, g_{\text{ref}}^t)$ *(reference similarity)*
  $f_3(g_k^t) \leftarrow \frac{1}{|S_t|-1}\sum_{j\neq k}\text{cosine}(g_k^t, g_j^t)$ *(peer consensus)*
  $f_4(g_k^t) \leftarrow \|g_k^t\|_2$ *(L2 norm)*
  $f_5(g_k^t) \leftarrow \frac{1}{d}\sum_{i=1}^{d}\mathbb{I}[\text{sign}(g_k^t[i]) = \text{sign}(g_{\text{ref}}^t[i])]$ *(sign consistency)*
  $\phi_k^t \leftarrow \text{MonteCarloShapley}(g_k^t, S_t, M)$ *(Shapley contribution)*
  $f_6(g_k^t) \leftarrow 0.8\phi_k^t + 0.2 f_6(g_k^{t-1})$ *(temporal smoothing)*
  $f_k^t \leftarrow [f_1, f_2, f_3, f_4, f_5, f_6](g_k^t)$
end for
Output: Fingerprint vectors $\{f_k^t\}_{k\in S_t}$

Algorithm S3: Trust-Weighted Aggregation with RL Adaptation

Algorithm 3 Trust Scoring and Aggregation (server-side, per round t)

Input: Gradients $\{g_k^t\}_{k \in S_t}$, fingerprints $\{f_k^t\}_{k \in S_t}$, DDQN networks $(Q_{\text{online}}, Q_{\text{target}})$, replay buffer $\mathcal{R}$
// **Dual Attention Processing**
for each client $k \in S_t$ **do**
    $\tilde{g}_k^t \leftarrow \text{Attention}(g_k^t)$ *(gradient embedding)*
    $\alpha_k^t \leftarrow \text{softmax}(W_f f_k^t)$ *(feature attention)*
    $h_k^t \leftarrow \sigma(W_g \tilde{g}_k^t + W_f(\alpha_k^t \odot f_k^t))$ *(joint embedding)*
    $\hat{w}_k^t \leftarrow \text{sigmoid}(u^\top h_k^t)$ *(prior trust weight)*
end for
// **RL-Based Refinement**
Form state: $s_t \leftarrow \{h_k^t\}_{k \in S_t}$
Select action: $a_t \sim \epsilon\text{-greedy}(Q_{\text{online}}(s_t))$
for each client $k \in S_t$ **do**
    $w_k^t \leftarrow 0.3 a_{t,k} + 0.7 \hat{w}_k^t$ *(blend RL action with prior)*
end for
// **Trust-Weighted Aggregation**
Normalize: $w_k^t \leftarrow w_k^t / \sum_{j \in S_t} w_j^t$
Aggregate: $\theta^{t+1} \leftarrow \theta^t + \sum_{k \in S_t} w_k^t g_k^t$
// **RL Policy Update**
Compute reward: $R_t \leftarrow \alpha \Delta \text{ACC}_t - \beta \text{FPR}_t - \gamma \text{FNR}_t$
Store: $\mathcal{R} \leftarrow \mathcal{R} \cup \{(s_t, a_t, R_t, s_{t+1})\}$
if $t \mod 10 = 0$ **then**
    Update Q_{online} via mini-batch from $\mathcal{R}$
    Soft-update: $Q_{\text{target}} \leftarrow 0.99 Q_{\text{target}} + 0.01 Q_{\text{online}}$
end if
Output: Updated global model θ^{t+1}

1.2 Monte Carlo Shapley Value Estimation (Detailed)

The Shapley value computation involves evaluating all possible permutations of client ordering, which is computationally intractable for large federations ($O(|S_t|!)$ complexity). We employ Monte Carlo approximation with adaptive sampling:

Adaptive Sampling Strategy:

- **Normal conditions** ($|S_t| \leq 10$, no anomalies detected): $M = 50$ permutations
- **Suspected attack** (outlier indicators triggered): $M = 200$ permutations
- **Large federations** ($|S_t| > 20$): $M = \max(50, 3|S_t|)$ for statistical stability

Permutation Evaluation Optimization:

1. **Incremental aggregation:** Maintain running aggregated model θ_{agg} as clients are added according to permutation order, avoiding recomputation from scratch.
2. **Cached validation:** Reuse validation loss computation when same subset of clients appears across permutations.
3. **Parallel processing:** Evaluate independent permutations across multiple CPU cores (8-16 cores typical).

4. **Early termination:** If Shapley estimate variance across first 30 permutations is below threshold (0.01), terminate early to save computation.

Wall-Time Analysis: On NVIDIA RTX 3090 with 16-core CPU, Shapley computation adds approximately 2-5 seconds per round for 10 clients with $M = 50$, scaling linearly with M and $|S_t|$.

1.3 Convergence Proof Sketch (Detailed)

Under the following assumptions:

1. **L-smoothness:** $\|\nabla F(\theta_1) - \nabla F(\theta_2)\| \leq L\|\theta_1 - \theta_2\|$ for all θ_1, θ_2
2. **Strong convexity:** $F(\theta_2) \geq F(\theta_1) + \nabla F(\theta_1)^\top(\theta_2 - \theta_1) + \frac{\mu}{2}\|\theta_2 - \theta_1\|^2$
3. **Bounded gradient variance:** $\mathbb{E}\|\nabla F_k(\theta) - \nabla F(\theta)\|^2 \leq \sigma^2$ for all k, θ
4. **Trust weight normalization:** $\sum_{k \in S_t} T(g_k^t) = 1$ and $0 \leq T(g_k^t) \leq 1$

The expected squared gradient norm at round $t + 1$ satisfies:

$$\begin{aligned}
\mathbb{E}\|\nabla F(\theta^{t+1})\|^2 \leq{}& (1 - \eta\mu)\mathbb{E}\|\nabla F(\theta^t)\|^2 \\
&+ \eta^2 L^2 \, \mathbb{E} \underbrace{\left\| \sum_{k \in S_t} \frac{|\mathcal{D}_k|}{|\mathcal{D}|} (\nabla F_k(\theta^t) - \nabla F(\theta^t)) \right\|^2}_{\text{heterogeneity variance} \leq \sigma^2} \\
&+ \eta^2 L^2 \, \mathbb{E} \underbrace{\left\| \sum_{k \in S_t} (T(g_k^t) - \frac{|\mathcal{D}_k|}{|\mathcal{D}|}) g_k^t \right\|^2}_{\text{Byzantine noise} = \Omega_{\text{Byz}}}
\end{aligned}$$

Byzantine Noise Term Analysis:

Under benign conditions ($\Omega_{\text{Byz}} = 0$), this reduces to standard FedProx convergence rate $O(1/T)$. Under Byzantine attack with trust weighting:

- **Worst case (no defense):** $\Omega_{\text{Byz}} \approx \rho \cdot \gamma^2 \sigma^2$ where ρ is malicious ratio and γ is attack intensity.
- **With OptiGradTrust:** Empirically, we observe $\Omega_{\text{Byz}} \leq 0.25\sigma^2$ even at $\rho = 0.4$, $\gamma = 20$, indicating effective Byzantine noise suppression via trust weighting.

Convergence Rate: Choosing learning rate $\eta \leq \frac{1}{2L}$ and assuming $\Omega_{\text{Byz}} \leq C\sigma^2$ for constant C, we obtain:

$$\mathbb{E}[F(\theta^T) - F(\theta^*)] \leq O\left(\frac{\sigma^2 + C\sigma^2}{\mu T}\right) + O\left(\frac{L\sigma^2}{\mu^2}\right)$$

demonstrating linear convergence to neighborhood of optimum with radius dependent on Byzantine noise level.

Table 1: **Supplementary Table S1: FedBN-P Masked vs Unmasked Proximal Term.** Evaluation on Alzheimer's MRI under Non-IID conditions (Dirichlet $\alpha = 0.5$) with scaling attack ($\times 10$) and 30% malicious ratio over 25 rounds.

Optimizer Variant	Test Accuracy (%)	Convergence Rounds
FedBN-P (masked, default)	95.80	24
FedBN-P (unmasked)	95.12	26
FedBN only	95.23	28
FedProx only	94.45	27

2 Supplementary Results

2.1 Extended Ablation: Optimizer Variants

Table S1 presents expanded optimizer ablation comparing FedBN-P masked (default) versus unmasked proximal term, evaluated under Non-IID (Dirichlet $\alpha = 0.5$) with 30% malicious clients.

Observation: Masked proximal (excluding BatchNorm from proximal term) achieves best accuracy and fastest convergence, validating the FedBN locality assumption for heterogeneous medical data.

2.2 Hyperparameter Sensitivity

Table S2 summarizes sensitivity analysis for key hyperparameters: proximal coefficient μ, RL blend factor τ, and Shapley smoothing β.

Table 2: **Supplementary Table S2: Hyperparameter Sensitivity Analysis.** Impact of varying key hyperparameters on Alzheimer's MRI accuracy under Non-IID (Dirichlet $\alpha = 0.5$) with 30% malicious clients (scaling attack $\times 10$).

Parameter	Value Range	Accuracy (%)	Comment
Proximal μ	0.001	94.23	Under-regularized
	0.01 (default)	95.80	Optimal balance
	0.1	93.67	Over-constrained
RL blend τ	0.1	95.12	Prior-dominated
	0.3 (default)	95.80	Balanced
	0.7	94.89	Action-dominated
Shapley β	0.5	95.34	High temporal variation
	0.8 (default)	95.80	Optimal smoothing
	0.95	95.56	Over-smoothed

Key Insight: Default hyperparameters ($\mu = 0.01$, $\tau = 0.3$, $\beta = 0.8$) are robust across $\pm 50\%$ perturbations, with accuracy degrading by ¡2% at extreme values.

2.3 Per-Class Performance Metrics

Table S3 provides detailed per-class diagnostic performance on Alzheimer's MRI, demonstrating balanced accuracy across disease severity stages.

Observation: OptiGradTrust maintains balanced performance across disease severity classes despite class imbalance (45% Non-Demented, 10% Moderate), validating the effectiveness of class-weighted cross-entropy loss.

Table 3: **Supplementary Table S3: Per-Class Diagnostic Performance on Alzheimer's MRI.** OptiGradTrust under IID conditions with 30% malicious clients (scaling attack $\times 10$). Metrics computed on test set (1,279 samples) after 25 federated rounds.

Disease Class	Precision (%)	Recall (%)	F1-Score (%)	Samples (test)
Non-Demented	98.2	97.5	97.8	575
Very Mild	95.3	94.1	94.7	384
Mild Demented	96.8	97.2	97.0	192
Moderate Demented	97.5	96.9	97.2	128
**Macro Average**	**96.95**	**96.43**	**96.68**	**1,279**

2.4 Computational Complexity Analysis

Table S4 provides detailed per-round computational complexity for each OptiGradTrust component.

Table 4: **Supplementary Table S4: Per-round Computational Complexity Summary.** Dominant asymptotic terms shown for OptiGradTrust components. Batched matrix operations (BLAS) and GPU parallelization reduce wall-time constants. Adaptive strategies (Shapley M, peer subsampling) maintain near-linear scaling with client count $|S_t|$.

Component	Complexity (per round)
Client local training (k)	$C_{\text{train}}(k) + O(E\,d)$ (prox penalty)
Server VAE recon (per client)	$C_{\text{VAE}}(d)$
Server cosine-to-ref (per client)	$O(d)$
Server peer-consensus (all clients)	$O(\lvert S_t\rvert\, d)$ batched (naive $O(\lvert S_t\rvert^2 d)$)
Server norms/sign (per client)	$O(d)$
Shapley (Monte Carlo)	$O(M\,\lvert S_t\rvert) \times C_{\text{val}}$ (parallelizable)
Attention embeddings (all clients)	$O(\lvert S_t\rvert\, P\, d)$
DDQN control	$O(\lvert S_t\rvert)$
Uplink per client	$O(d/Q)$ bits (with quantization)

2.5 Attack Intensity Robustness

Table S5 summarizes framework performance under varying Byzantine attack intensities.

Table 5: **Supplementary Table S5: Robustness Under Varying Attack Intensity.** Final test accuracy (%) on Alzheimer's MRI classification as Byzantine attack intensity (γ, scaling factor) increases from 5× to 40× with 40% malicious client ratio over 25 federated rounds. OptiGradTrust maintains ¿91% accuracy even at extreme intensity (40×), demonstrating graceful degradation compared to sharp drops in baseline methods.

**Method**	**γ=5**	**10**	**20**	**40**
FedAvg	94.2	91.8	89.0	83.6
FLTrust	94.6	92.9	90.5	85.1
OptiGradTrust (Ours)	95.1	94.3	93.7	91.2

3 Supplementary Discussion

3.1 Comparison with FedPxN-Style Hybrids

While Hwang et al. (2023) proposed FedPxN combining FedBN and FedProx for medical federated learning, their focus was optimization stability under statistical heterogeneity in honest-client scenarios. OptiGradTrust's FedBN-P differs in three key aspects:

1. **Adversarial co-design:** FedBN-P is integrated with trust-aware aggregation, so the optimizer's proximal term complements RL-driven trust weighting rather than operating independently.

2. **Masked proximal formulation:** We explicitly partition parameters into $\mathcal{B}$ (BatchNorm) and $\bar{\mathcal{B}}$ (non-BN), applying proximal regularization only to $\bar{\mathcal{B}}$, whereas FedPxN applies standard FedProx globally.

3. **Byzantine-aware hyperparameter tuning:** Our $\mu = 0.01$ is tuned under adversarial conditions (30-40% malicious clients), whereas FedPxN optimizes for convergence speed in benign settings.

These distinctions result in FedBN-P achieving superior accuracy-robustness trade-offs specifically under Byzantine attacks, as demonstrated in our ablation studies.

3.2 Limitations of Synthetic Alzheimer's Dataset

The Kaggle Alzheimer's MRI dataset used in this study is a synthetic, publicly released collection designed for educational purposes. Key limitations include:

- **Artificial data generation:** Images are synthetically generated rather than real patient scans, lacking realistic artifacts (motion blur, scanner noise, patient-specific anatomical variations).

- **Simplified pathology:** Disease progression is categorized into discrete stages (Non-Demented, Very Mild, Mild, Moderate) without longitudinal trajectories or subtle biomarkers.

- **Homogeneous acquisition:** All scans follow identical synthetic protocol, missing real-world heterogeneity from 1.5T vs 3T scanners, varying slice thickness, different acquisition sequences (T1, T2, FLAIR).
- **Limited sample size:** 6,983 samples is small compared to clinical research datasets (ADNI: 10,000+ scans, UK Biobank: 100,000+ participants).
- **No demographic diversity:** Lacks representation of age, sex, ethnicity, comorbidities that affect real diagnostic models.

Implications for Results: Our reported accuracies (96.61% average, 96.92% best case on synthetic MRI) likely overestimate performance on real clinical data. Validation on gold-standard datasets (ADNI, FeTS, UK Biobank) is essential before clinical deployment claims can be substantiated.

3.3 Comparison with Clinical FL Benchmarks

Recent clinical federated learning studies report:

- **FeTS (Federated Tumor Segmentation):** 23 institutions, Dice coefficient 0.86 for glioblastoma segmentation (Sheller et al., 2019).
- **Multi-center Alzheimer's diagnosis:** 5 institutions, 85% accuracy on real longitudinal ADNI data (Islam et al., 2023).
- **COVID-19 chest X-ray:** 10 hospitals, 92% AUC under data heterogeneity.

Compared to these benchmarks, OptiGradTrust's 96.61% average accuracy (96.92% best case) on synthetic data is encouraging but not directly comparable. Our framework's value lies in Byzantine robustness mechanisms (six-signal fingerprinting, RL-driven trust) rather than raw accuracy on toy datasets.

4 Supplementary References

Extended bibliography for methods and datasets referenced in supplementary materials is included in the main manuscript reference list.
